# Electrochemical Biosensors for Cancer Diagnosis and Prognosis Using Protein Biomarkers: Current Trends, Advances, and Clinical Translation Potential

**DOI:** 10.3390/s26041139

**Published:** 2026-02-10

**Authors:** Michael E. J. López Mujica, Elena E. Ferapontova

**Affiliations:** Interdisciplinary Nanoscience Center (iNANO), Faculty of Natural Sciences, Aarhus University, Gustav Wieds Vej 1590-14, DK-8000 Aarhus, Denmark; mlopezm@inano.au.dk

**Keywords:** electrochemical biosensors, aptasensor, immunosensor, protein biomarkers of cancer

## Abstract

Cancer, a disease with high mortality, represents a major public health challenge. Increased access to early tumor screening, especially non-invasive liquid biopsy assays targeting blood-circulating protein biomarkers, has advanced cancer diagnosis and treatment, but these assays are still scarce. This work critically reviews general strategies for the rapid and accurate electrochemical detection of serum proteins and surveys recent advances in liquid biopsy electrochemical biosensors targeting cancer-related proteins. Many of these approaches have achieved remarkable analytical sensitivity. The review further addresses key barriers to clinical translation and commercialization, including complex sample matrix effects that require rigorous standardization of preanalytical and analytical workflows, limited validation using patient samples, difficulties in accounting for interpatient variability, and practical considerations such as manufacturability, cost-effective scale-up, and long-term stability. Accordingly, particular emphasis is placed on clinically translatable detection methods, with a focus on the analytical and clinical validation of biosensors.

## 1. Introduction

**Figure 1:**
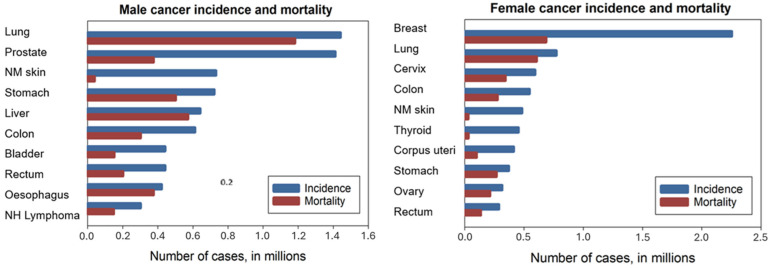
Gender-specific cancer incidence and mortality worldwide in 2020, according to [2]. Data for ten most frequently diagnosed cancers are shown, NM: non-melanoma; NH: non-Hodgkin.

During the COVID-19 pandemic and the years that followed, bioanalytical research increasingly shifted toward emergency diagnostics, driving the development of numerous advanced systems and devices for the rapid and accurate detection of infectious diseases and, ultimately, chronic conditions such as cancer. The ongoing cancer pandemic [1] takes approximately 10 million lives each year, and more than 19 million new cases are diagnosed annually, including 10.1 million male and 9.2 million female cancers [2]. The global burden is expected to intensify, as populations age, with projections estimating 28.4 million new cancer cases by 2040 [3]. Therewith, gender-specific prostate and breast cancers, along with lung cancer, are in the top list of most frequently detected, and both mortality and survival rates for different types of cancer differ significantly (Figure 1). For prostate and breast cancer, for which wide-population screening programs are running, the one-year survival rates are between 97–99% and 96–98% (depending on the country), correspondingly, while for lung cancer, the one-year survival rate is only 41–50%, further dropping to 17–21% in the case of the five-year survival rate [4]. Similarly low five-year survival rates are observed for other cancers characterized by internal tumors: 6% 5-year survival rate for pancreatic cancer or 13% for esophageal cancer, which can be attributed, among other reasons, to the absence of diagnostic tools for early detection of internal tumors and their aggressiveness [5]. Most patients enter the healthcare system through pathology-based diagnoses, as both primary and recurrent cancers are still predominantly identified from tissue samples [6]. This process is time-consuming and often results in cancers, particularly those characterized by internal, hard-to-access tumors, being detected at relatively late stages or misdiagnosed, thereby limiting treatment success.

Over the past few decades, significant advances in cancer diagnosis and treatment have been achieved as a result of earlier detection of tumors, new treatments available, greater availability of screening tests, and safer forms of chemotherapy with reduced complications [7]. Greater availability of screening tools for early tumor detection, including non-invasive diagnostic tools, such as magnetic resonance imaging (MRI) and liquid biopsy analysis of blood-circulating tumor biomarkers, undeniably contributed to the progress achieved with some cancers’ diagnosis and treatment. MRI is not a technique suitable for general population-wide screenings, and liquid biopsy-based analysis of circulating cancer biomarkers released by tumor cells into biological fluids, such as blood, plasma, serum, saliva, urine, and gastric juice, is more suitable for timely detection of cancer through wide-population screening programs. However, only a limited number of liquid biopsy tests has been approved by the FDA for guiding clinical cancer analysis: a serum prostate-specific antigen (PSA) test for prostate cancer and a serum alpha-fetoprotein (AFP) test for liver cancer detection, both tests suffering from false positives rates, since both proteins may be elevated in non-malignant conditions [8]. These two tests only indicate the need for further investigation, and cancer must still be confirmed by invasive solid biopsy. This highlights a major hurdle in the clinical implementation of liquid biopsy: the lack of accurate and adaptable tools for the clinical validation of serum cancer biomarkers, which are typically identified through studies of their expression in tumor tissue. The critical question, therefore, remains: what should be detected in blood to enable early cancer diagnosis while avoiding both underdiagnosis and overdiagnosis?

Another important question is regarding which bioanalytical platform can enable both the discovery of reliable liquid biopsy biomarkers for a specific cancer or its stage and support their accurate and timely detection. Among the many available platforms, electrochemical detection approaches stand out as the fastest, most cost-effective, and user-friendly [9]. Compared with optical platforms widely used in clinical settings, which rely on bulky instrumentation, require precise optical alignment, and often depend on transparent sample paths, electrochemical platforms are more tolerant to complex sample matrices. They combine high analytical sensitivity with compact, low-power instrumentation that can be readily integrated into portable readers and disposable electrode formats, thereby simplifying miniaturization and reducing overall cost. In addition, electrochemical approaches enable straightforward integration with microfluidics and multiplexed electrode arrays, facilitating rapid analysis from small sample volumes and supporting quantitative measurements across clinically relevant concentration ranges. Consequently, electrochemical biosensors show great promise for the development of inexpensive, high-performance testing devices for point-of-care (POC) applications. Such devices should be largely autonomous, independent of specialized laboratory personnel or complex facilities, and capable of delivering quantitative results within the duration of a general practitioner visit [10]. While optical methods provide excellent performance in centralized laboratory settings, their translation to decentralized POC formats is often limited by higher instrument complexity and cost as well as sensitivity to matrix-related interferences (e.g., turbidity, scattering, or autofluorescence) in real-world biofluid samples [11]. Yet, despite global progress, cancer-targeting POC testing devices remain very limited, constrained by high costs that restrict accessibility for large populations and by their frequent inability to reliably detect diseases at early stages [12].

In this work, we discuss the capabilities of electrochemical biosensors and, based on an analysis of existing studies on serum-circulating protein biomarkers, examine how these biosensors address two central challenges in cancer diagnostics: what to detect for effective cancer identification and how to detect it. Here, we focused primarily on recent biosensor platforms; electrochemical aptasensors for cancer diagnostics published before 2021 were comprehensively addressed in our earlier work [9].

## 2. Requirements for Accurate Biosensors and Receptors Used in Their Design

Liquid biopsy assays designed to detect tumor-derived biomarkers circulating in blood constitute a promising non-invasive strategy for cancer diagnosis and therapeutic monitoring. Yet, in early cancer detection, any liquid biopsy screening can be regarded as a tumor-uninformed approach. To address the challenge of what to detect in blood for the early and accurate identification of cancer, attention inevitably turns to tumor-specific biomarkers released into the bloodstream by cancer cells and tissues [13], which can include:

○ Circulating tumor cells (CTCs), which are rare intact malignant cells released from primary and/or metastatic lesions into the bloodstream. CTC enumeration and molecular profiling (e.g., epithelial/mesenchymal markers, mutation status, therapy-response signatures) provide prognostic information and enable longitudinal monitoring of tumor evolution [14].
○ Proteins, shed or secreted by tumors and the tumor microenvironment, including cytokines, growth factors, and tumor-associated antigens. Representative examples include PSA (prostate cancer), CA 19-9 (pancreatic cancer), CEA (colorectal and other cancers), AFP (hepatocellular carcinoma), and CA-125 (ovarian cancer) [15].
○ Cell-free DNA (cfDNA), which consists of 100–180 base pair (bp) DNA fragments originating from multiple sources, of which circulating tumor DNA (ctDNA) constitutes only a small fraction (up to 10%), in cancer patients’ samples [16,17].
○ MicroRNAs, which are endogenous, small (~18–25 nucleotides) non-coding RNAs that regulate gene expression post-transcriptionally by binding target mRNAs. In cancer, dysregulated microRNA expression reflects key oncogenic processes (proliferation, invasion, EMT, drug resistance). Circulating microRNAs originate from tumor cells and surrounding tissues and are present in biofluids either bound to proteins (e.g., Argonaute complexes) or packaged within lipoproteins and extracellular vesicles, which enhances their stability and makes them attractive liquid biopsy biomarkers [18,19].
○ Extracellular vesicles (EVs), which are membrane-enclosed nanoparticles secreted by the majority of cell types, including tumor cells, and widely distributed across biofluids such as blood, urine, and saliva. EVs carry tumor-informative cargo: proteins, lipids, metabolites, DNA, mRNA, and microRNAs, which reflect the molecular state of the originating cells. Because EVs protect their molecular cargo from degradation and can be selectively enriched using surface markers (e.g., epithelial cell-adhesion molecules and the tetraspanin proteins CD63 and CD81), they are highly relevant for cancer diagnostics, disease stratification, and monitoring [20,21].
○ Tumor-derived metabolites, which are low-molecular-weight metabolic products altered by tumor metabolism and/or the tumor microenvironment and detectable in biofluids. Examples include lactate (enhanced glycolysis/Warburg effect), 2-hydroxyglutarate (IDH-mutant tumors), sarcosine (reported in prostate cancer), choline-containing metabolites (membrane turnover), and selected amino acid and TCA-cycle intermediates. Metabolite panels can complement genomic and proteomic markers by reporting functional and dynamic changes in tumor biology [22,23].

To improve cancer diagnosis and treatment, the biomarker must provide accurate information about cancer status and help avoiding both overdiagnosis and underdiagnosis. Progress in this field strongly correlates with the availability of biofluid-circulating biomarkers amenable to sensing, their robustness and clinical relevance for cancer detection, and the existence of suitable receptors and analytical platforms for their specific detection. In early-stage cancer, liquid biopsy testing can be particularly challenging due to the low abundance of some potential biomarkers, especially when tumor volumes are very small (on the order of a few mm^3^). For example, in advanced cancer patients, circulating tumor DNA (ctDNA) levels in a milliliter of blood can reach hundreds of genome copies, typically present as 100–200 bp fragments. Yet, in early-stage cancers, the median concentration of ctDNA drops 1000-fold, and, considering that ctDNA coexists with a large excess of non-mutated cfDNA (billions of fragments), ctDNA detection becomes very challenging [16].

This review focuses on recent advances in the analysis of serum-circulating proteins whose overexpression in tumors has been associated with various cancer types (Table 1). Although these proteins are upregulated in tumor cells, they are also expressed in healthy tissues, which may result in their relatively high basal concentrations in biofluids, falling in the sub-picomolar to picomolar range. It is important to stress that only a few of these proteins are approved by FDA for liquid biopsy detection of cancer. Biosensors for protein cancer biomarkers should, therefore, exhibit not only high detection specificity (to avoid false positives), appropriate limits of detection (to avoid false negatives), sufficient analytical speed, and good storage stability but also the accuracy required for clinical validation of liquid biopsy assays targeting these proteins. Therewith, tumor heterogeneity (between patients, between lesions, and within a single lesion) can “erode” the specificity of a single serum/plasma protein biomarker for cancer diagnosis in a few recurring ways:

- Non-uniform protein expression and secretion across tumor clones/regions gives inconsistent “tumor signal” in blood. If only some subclones (or tumor areas) express/secret a candidate protein, the circulating level can be intermittent or low, increasing overlap with noncancer controls and weakening specificity. Intratumor heterogeneity and branched evolution are, thus, widely recognized obstacles for validation of any biomarker that assumes a single, stable tumor state [24,25,26].
- Temporal heterogeneity (evolution over time or due to treatment pressure) can result in cancer signatures drifting. As tumors evolve, the dominant clones and their secreted proteins can change, so a marker that looked “specific” at one time point may become less discriminative later, and vice versa [25,26].
- Microenvironment and host-response proteins contaminate the signal which results in cancer versus, e.g., inflammation, becoming hard to separate. Many circulating proteins reflect immune activation, stromal remodeling, or systemic inflammation rather than tumor-exclusive biology; those processes also occur in benign disease, infection, obesity, autoimmune conditions, etc., which increases false positives and reduces specificity [27,28,29].
- Secretome heterogeneity and dilution in blood mask low-abundance tumor-derived proteins. Tumor-secreted/shed proteins enter a high-dynamic-range background (abundant plasma proteins) and are additionally shaped by clearance and peripheral production. Heterogeneity in what the tumor secretes amplifies this problem and can push distributions of cases and controls to overlap [28,29,30].
- Finally, “single-biomarker” logic breaks in heterogeneous early disease can result in poor apparent specificity in real-world screening. It is increasingly argued that protein biomarker tests need higher dimensionality (panels, multi-omics, machine-learning models) because heterogeneity makes single proteins insufficiently specific and robust [31,32,33].

The loss of specificity of single serum protein cancer biomarkers caused by tumor heterogeneity can be addressed in several ways, including, as already mentioned, the use of multimarker panels and machine-learning-based pattern analysis and longitudinal monitoring and sensitive multiplexed platforms with advanced assays [34], the latter being a primary focus of current biosensor research.

**Figure 2:**
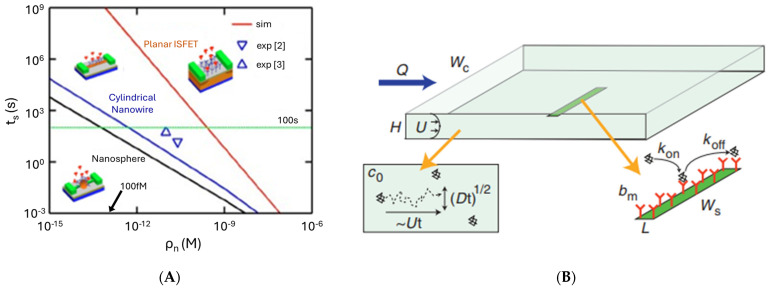
(**A**) Tradeoff between the settling time and detectable concentration [36] and (**B**) model system studied in [37]: Solution with target concentration *c*_0_ flows with velocity *U* and volumetric flow rate *Q~HW_c_U* through a channel of height *H* and width *W*_c_ over a sensor of length *L* and width *W*s that is functionalized with *b*_m_ receptors per unit area. The kinetic rate constants for the (first order) binding reaction are *k*_on_ and *k*_off_, and the diffusivity of the target molecules is *D* [37]. Reproduced with permission from ACS and Springer.

In this context, an ideal biosensor design should account for high antifouling resistance, as analyses are performed in complex matrices such as serum or whole blood [35]. The typically low concentrations of target proteins in physiological fluids, which may range from the femtomolar to picomolar level under basal expression and increase to the sub-nanomolar–nanomolar level in cancer, impose additional constraints on biosensor performance characteristics. Diffusion limitations can increase macrosensor response time to unreasonable times, and the use of micro- and nanoscale electrodes, along with a variety of surface micro- and nanostructuring strategies, is widely considered an effective approach to overcome this limitation (Figure 2A, theoretical predictions) [36]. At low analyte concentrations, bioreceptors may also exhibit different affinities and binding kinetics, which can be optimized to improve both sensing performance and sensor regeneration (Figure 2B) [37]. In particular, mass transport by diffusion and convection controls the local analyte concentration at the sensor surface and, therefore, the effective association rate, while receptor surface density (*b*_m_), immobilization chemistry, and sensor geometry determine how many binding events can occur and whether equilibrium binding is observable. In addition, electrostatic interactions and buffer conditions can significantly enhance association kinetics without altering the intrinsic dissociation constant. Keeping all these in mind, apparent binding affinities can be deliberately modulated, for example, through sensor design and fluidic conditions (flow velocity *U*/volumetric rate *Q* and geometry and size of the channel through which the sample flows) and surface functionalization rather than by changes in biomolecular recognition alone [37]. In addition, the interplaying surface density and conformational state of bioreceptors can jointly affect their availability for ligand binding, and, thus, the overall sensor performance and should, therefore, be carefully controlled [38].

**Figure 3:**
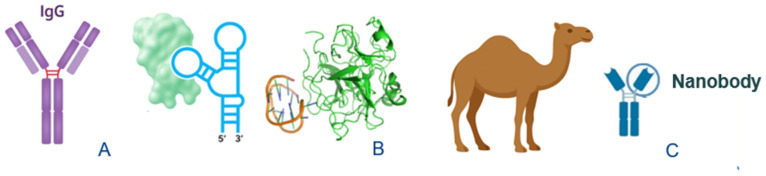
Examples of bioreceptors most widely used in design of biosensors for protein targets: (**A**) antibodies (immunoglobulin G, the most abundant antibody type in blood and extracellular fluid, marked in violet); (**B**) aptamers (represented in blue and sandy brown) interacting with proteins (shown in green); and (**C**) nanobodies, which are isolated variable domains of the heavy chain of the heavy-chain-only camelid antibodies (shown in blue), marked by blue circle.

Accordingly, the design of protein biosensors directly depends on the affinity interactions between the bioreceptor and the target protein and, thus, on the availability of an appropriate bioreceptor. The bioreceptors, or biorecognition elements, most commonly used at present, include antibodies and aptamers as well as the more recently emerging nanobodies (Figure 3). Monoclonal antibodies, widely used for the bioanalysis of protein biomarkers of cancer [39,40], often exhibit limited lot-to-lot reproducibility in performance and efficacy, which is inconsistent with their high cost [41,42]. An attractive alternative to antibodies, whose production relies on human or animal source, is the use of aptamers, often referred to as nucleic acid antibodies [43,44] (note that peptide aptamers also exist, though they remain a minority). Aptamers are produced and modified in vitro using automated protocols, making them a cost-effective option that facilitates the design of biosensors for cancer-related proteins [9]. Demonstrating comparable binding affinities in the pM–nM range, aptamers offer tunable aptamer–ligand interactions that can be modulated by the surrounding medium and temperature, and they are straightforward to chemically modify. Also, aptamers generally exhibit enhanced chemical stability and greater resistance to environmental degradation, making them even more attractive alternatives in situations where antibody denaturation or instability in complex media, due to proteolytic degradation, fouling, their temperature, pH, and repeated wet–dry cycles sensitivity, is problematic [45]. These well-documented stability limitations of antibodies, including susceptibility to proteolysis and aggregation throughout production, formulation, and exposure to biological matrices, can impair their functional performance in untreated serum, with degradation occurring on timescales of hours to days [46]. For example, in serum, immunoglobulin G (IgG) molecules may undergo proteolytic cleavage, aggregation, or conformational changes, which can compromise their binding potency and analytical reliability [47]. In contrast, aptamers are much more stable than antibodies, and their susceptibility to nuclease digestion (particularly, of RNA aptamers) can be successfully combatted by chemical modifications (e.g., 2′-fluoro, 2′-O-methyl, locked nucleic acids), extending half-life in human serum from seconds to hours or longer depending on the modification strategy [48,49]. Modified and, importantly, immobilized aptamers can remain functional for days to weeks in serum; they are suited for continuous monitoring and regeneration [35,50] and are more tolerant to temperature and storage variations.

Nanobodies (12–15 kDa), derived from the single-variable domains of camelid antibodies, represent another promising and cost-effective alternative to conventional antibodies, as their engineering and expression in bacterial systems are simpler and more economical [51,52]. Owing to their smaller size, exceptional stability, and reduced structural complexity compared with bulky ~150 kDa full-length antibodies, nanobodies can be readily modified using site-specific labeling strategies that preserve binding-site affinity, often compromised by random conjugation protocols used with antibodies [53]. For both aptamers and nanobodies, multimerization has emerged as a popular strategy to enhance avidity and target specificity for in vivo applications and in vitro sensing [54,55] and holds significant potential for enabling the development of high-performance biosensors.

## 3. Basic Electrochemical Platforms for Electrochemical Detection of Proteins

**Figure 4:**
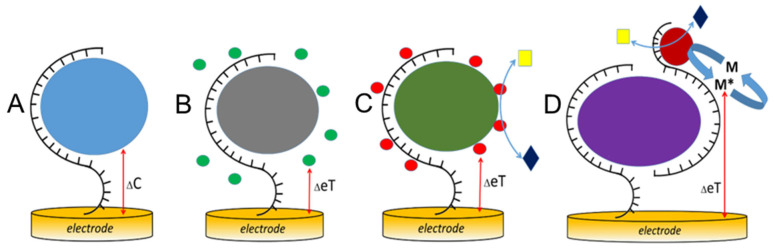
Schematic illustration of the fundamental principles of electrochemical protein biosensing (large circles denote proteins) using aptamers immobilized on electrodes (similar principles apply to immunosensing): (**A**) capacitive sensing; (**B**,**C**) redox indicator-based detection schemes, green circles denote a soluble redox indicator; red circles: a redox indicator adsorbed on the protein and aptamer surface; diamond and square: another redox indicator whose redox transformation is catalyzed by the “red” one; and (**D**) electrochemical sandwich ELISA, the purple circle: an enzyme or nanozyme label conjugated to the reporter aptamer; diamond and square: enzyme substrate and the product of catalysis, and M/M*: redox mediator shuttling electrons between the electrode and the label, unless the label directly communicates with the electrode.

Once a specific protein biomarker has been selected, the electrochemical platform must enable robust and accurate analysis of this target (Figure 4). Accordingly, the binding of bulky and charged protein targets to their receptors immobilized on electrode surfaces induces significant changes in the electrical double layer leading to changes in electrode capacitance, which can be detected by capacitive sensing (Figure 4A). Label-free capacitive sensing allows the development of inexpensive yet high-performance POC testing devices based on disposable but efficient capacitive electrode systems [56]. Capacitive sensing does not require any labeling reagents, often enabling rapid, one-step detection schemes. An exception is the recently introduced redox-capacitive sensors [57,58], which nonetheless typically demonstrate modest limits of detection (LODs). Alternatively, protein binding can be monitored via Faradaic signal variations in the presence of redox indicators (e.g., ferricyanide, methylene blue, ruthenium hexamine). These redox indicators present in solution may (1) interact differently with free receptors versus receptor–protein complexes and (2) exhibit hindered electrochemical responses following protein binding to the modified electrode surface (Figure 4B). This approach also generally results in modest LODs. Affinity-based bioreceptors typically operate through 1:1 binding interactions, and that, as a matter of fact, results in inherently low sensor responses at low analyte concentrations. Therefore, signal amplification strategies, most commonly exploiting electrocatalysis, are often required to achieve adequate sensitivity (Figure 4C,D). In electrocatalytic detection schemes, the signal can be amplified either through the use of an additional redox indicator, whose electrochemical transformation is electrocatalytically enhanced by the primary indicator that interacts, electrostatically or through other mechanisms, with the bioreceptor and the bioreceptor–protein complex (Figure 4C), or by employing (bio)electrocatalytic labels in, for example, sandwich immunoassay- or aptamer-based formats (Figure 4D).

Electrochemical biosensing approaches based on magnetic beads form a somewhat separate category. These methods predominantly involve electrochemical enzyme-linked immuno- and aptamer sorbent assays (e-ELISA and e-ELASA), employing both traditional ELISA-type redox enzyme labels, such as horseradish peroxidase, alkaline phosphatase, and glucose oxidase cascades, and less conventional hydrolase labels, such as cellulases [59]. The use of magnetic beads enables not only sample preconcentration and efficient bioseparation but also the reduction in matrix interference effects and, consequently, biofouling. E-ELISA/e-ELASA performed on magnetic beads is rapid, ultrasensitive, and often more specific than conventional optical ELISA for both genetic targets [60,61] and protein profiling [34,35].

## 4. Basic Electrochemical Techniques Used for Electrochemical Biosensing

The choice of electrochemical techniques for biosensing generally depends on the intended application. This includes whether interfacial processes associated with biorecognition are being studied, whether the proposed biosensor design is evaluated in model systems, or whether POC diagnostic devices are developed based on these models, for example, for liquid biopsy applications, where robust performance in complex matrices and straightforward signal acquisition and interpretation are critical [62].

**Figure 5:**
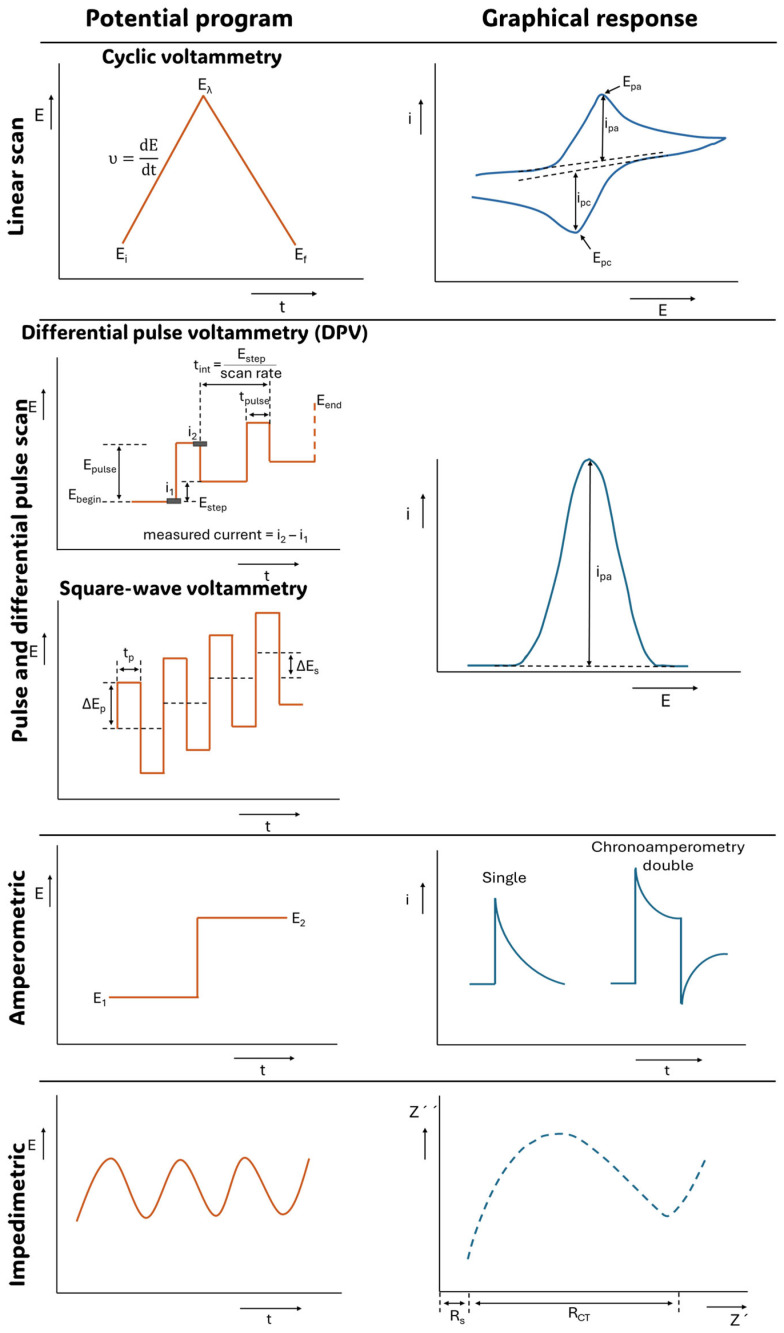
Schematic representation of applied potential programs (**left panels**) and typical signals (**right panels**) for five mainstream electrochemical techniques: cyclic voltammetry, differential pulse and square-wave voltammetry, amperometry, and electrochemical impedance spectroscopy. Abbreviations: *E*: the applied potential, *t*: the pulse time; *E*_i_: the initial applied potential, *E*_λ_: vertex potential, where the scan direction is inverted; ν: the scan rate, *E*_pa_ and *E*_pc_: the anodic and cathodic peak potentials; *i*_pa_ and *i*_pc_: anodic and cathodic currents; *E*_begin_: the starting potential; *E*_end_: the ending potential; *E*_step_: the potential step (step increment); *E*_pulse_: the pulse amplitude, *t*_pulse_: the pulse duration (pulse width); *t*_int_: the time interval per step (time between consecutive staircase levels); *i*_1_ and *i*_2_: the currents sampled at two defined times; Δ*E*_s_: the step increment (staircase step potential); Δ*E*_p_: the square-wave amplitude (pulse amplitude); *t*_p_: the pulse time (half-period duration; the time spent at each level of the square wave before switching polarity); *E*_1_ and *E*_2_: the applied initial and final potentials; Z′: the real part of the impedance; Z″: the imaginary part of the impedance; *R*_s_: the solution resistance; *R*_CT_: the charge-transfer resistance.

In this context, electrochemical techniques can generally be grouped into: (i) linear potential sweep (voltametric) methods, (ii) pulse and differential pulse methods, and (iii) electrochemical impedance spectroscopy (EIS). Figure 5 summarizes the most popular electrochemical techniques regarding the applied potential waveform and the corresponding signal response [63]. In biosensors, voltammetry is often used to characterize interfacial electrochemistry and kinetics/potential window of the electrode reactions proceeding, particularly, when (bio)electrocatalysis is involved. Pulse techniques and differential pulse techniques, which account for background signals, are useful for more sensitive quantification, while EIS is widely adopted for label-free detection and monitoring surface binding and fouling.

**Voltammetry**, such as cyclic voltammetry (CV) and linear sweep voltammetry (LSV), measures the current while the potential is driven through a controlled trajectory. The current may contain a faradaic component associated with redox reactions and a non-faradaic component associated with charging of the electric double layer and dielectric layers (common for biofunctionalized electrodes). Voltametric analysis of redox-active processes aims to maximize the faradaic signal and minimize the capacitive background [64] and vice versa in capacitive sensing approaches. In biosensor research, CV and LSV are often used to confirm surface modification (via changes in peak currents and separations), reveal interfacial blocking or fouling, and validate the performance of redox tags and mediators. For quantitative detection in complex biofluids, however, CV is less useful, as substantial capacitive currents at coated or nanostructured electrodes can obscure small faradaic signals. Also, due to its dynamic nature, dependence of the characteristic parameters on the scan rate, and the necessity of signal background correction, CV and LSV readout and interpretation are not straightforward for untrained personal.

**Pulse voltametric techniques**, such as differential pulse voltammetry (DPV) and square-wave voltammetry (SWV), are designed to suppress background currents and are better suited for quantitative biosensing, enabling more sensitive detection in the systems relying on redox mediators and enzymatic products or surface-tethered labels (e.g., methylene blue or ferrocene on aptamers/DNA probes). In both techniques, the current is sampled at time points where capacitive charging currents have largely decayed, whereas the faradaic currents associated with redox processes persist. By exploiting this difference in time constants, pulse methods strongly enhance the signal-to-background ratio [65]. In DPV, the baseline scan proceeds as a staircase, and a short pulse is superimposed at each step. In each potential step, two current values are sampled, one near the end of the baseline period and the other near the end of the pulse, and the reported signal is taken as their difference. The resulting characteristic peak-shaped response is commonly used for calibration, with the peak height (or area) increasing as a function of analyte concentration. DPV typically provides excellent sensitivity in complex matrices and yields stable quantitative metrics that are straightforward to implement in POC-oriented devices [66]. Square-wave voltammetry (SWV) also employs a stepwise potential scan but replaces the single pulse with a symmetric square-wave modulation. SWV can be viewed as a rapid sequence of forward and reverse potential perturbations around each baseline level, where the current is sampled at the end of each half-cycle and the net response is obtained by subtracting the reverse current from the forward current. This forward–reverse differencing further suppresses capacitive contributions, as charging currents are typically similar in both directions, whereas faradaic currents depend strongly on the redox state and the direction of the applied perturbation. The outcome is, again, a peak-shaped response used for quantification; however, SWV can achieve this with shorter acquisition times than DPV, as it can be operated at higher effective frequencies. That speed makes SWV most perspective for rapid quantifications in portable platforms. The main practical limitation is kinetic: when electron transfer (ET) or mass transport is slow, overly high frequencies can distort the signal or reduce sensitivity, requiring SWV parameters to be matched to the system’s electrochemical timescale [67].

**Amperometry**, in turn, monitors the current as a function of time at a fixed applied potential, whereas chronocoulometry measures the total charge passed, corresponding to the amount of electricity involved in the electrochemical process. Accordingly, either a decay or an accumulation of the signal is recorded. In amperometry, when the potential is stepped to a value at which a redox reaction occurs, the current initially exhibits a sharp increase and then decays over time due to the depletion of redox-active species near the electrode surface. In chronocoulometry, the charge passed is monitored as a function of time, resulting in a cumulative increase that generally reflects the total amount of redox-active species consumed at the electrode surface or the charging of the electric double layer. Owing to their simplicity and ease of interpretation, both techniques are widely used in biosensing and are highly suitable for POC device operation. At the same time, they can suffer from baseline drift and electroactive interferents at the chosen potential, making detection potential selection critical for measurements in complex biofluids [68].

**Impedimetric approaches** probe the interface in the small-signal regime (small-amplitude AC perturbation, typically 5–10 mV) and separate interfacial processes by their characteristic time constants across frequency. Electrochemical impedance spectroscopy (EIS) is widely used in biosensing because it enables label-free monitoring of surface modification and biorecognition events: the formation of a biomolecular layer and subsequent target capture can alter the charge-transfer resistance (*R*_CT_) and interfacial capacitance, leading to measurable changes in impedance. This sensitivity to nanoscale interfacial structure makes EIS particularly well suited for monitoring surface functionalization quality, evaluating antifouling performance, and detecting bulky targets (e.g., proteins, extracellular vesicles, cells) that strongly perturb interfacial charge transfer and mass transport [69]. EIS data can be presented in several formats, including Nyquist plots (imaginary versus real impedance), Bode plots (impedance magnitude and phase versus frequency), and complex plane phase or angle representations. Among these, Nyquist plots are most commonly used in biosensor research because they provide an intuitive visualization of key interfacial elements, particularly the semicircular feature associated with *R*_CT_ and double-layer capacitance, enabling rapid comparison of surface modifications and target-binding events. Interpretation of EIS data relies on circuit-based or phenomenological models that translate impedance spectra into parameters such as *R*_CT_ and capacitive elements; however, strong model dependence and longer measurement times can become limiting when broad frequency ranges are required. For POC translation, these limitations are commonly mitigated by simplifying data acquisition, by using selected frequencies or shortened scans, and by applying robust calibration strategies to convert impedance features into clinically meaningful readouts [70].

Together, all these electrochemical methods strike a balance between the level of mechanistic insight they provide, their analytical sensitivity, and their operational simplicity. For POC cancer diagnostics, there is rarely a single “best” technique—selection mainly depends on the assay format (label-based or label-free), the type and expected level of the biomarker, and practical constraints such as speed, robustness, and performance in real samples.

## 5. Electrochemical Assays for Specific Proteins Detection

### 5.1. HER-2/neu

Human epidermal growth factor receptor 2 (HER-2/*neu*, or ErbB2) is a 185 kDa transmembrane glycoprotein belonging to the epidermal growth factor receptor family and plays a crucial role in cellular signaling pathways [71]. HER-2/*neu* is overexpressed in several aggressive cancers, including breast and esophageal cancers, being itself the target of anticancer therapy, and its overexpression in tumor cells often correlates with rapid tumor progression and poor prognosis. In the bloodstream, HER-2/*neu* circulates as its extracellular domain (ECD), which is proteolytically cleaved from the cell surface. It has, therefore, been proposed that serum HER-2/*neu* can be used for diagnosis of HER-2–positive cancers and for monitoring the treatment response [72]. Yet this suggestion still lacks consistent clinically validated correlations between tumor status and serum ECD levels.

Nevertheless, several basic electrochemical approaches for analysis of serum HER-2/*neu* were explored, including electrochemical immunoassay with ferricyanide as a redox indicator (Figure 2B), voltametrically detecting down to 0.14 pM HER-2/*neu* in 5% serum in 35 min [73] and electrochemical aptamer assay detecting from 1 pM to 10 nM HER-2/*neu* in 1% serum in 30 min [74]. In both cases, polyethylene glycol (PEG) was used to prevent electrode biofouling. In the first case, iron oxide nanoparticles modified with antibodies and PEG were deposited on gold nanoparticulated surface, additionally blocked with bovine serum albumin (BSA) [73]. In the second case, a thiolated aptamer and thiolated PEG co-immobilized on gold provided not only biorecognition interface but also a general electrocatalytic platform: PEG slowed down electrochemical transformation of ferricyanide on PEG-modified surface, which was then electrocatalytically amplified by methylene blue, electrostatically binding differently to the aptamer and the aptamer-protein complex [74].

**Figure 6:**
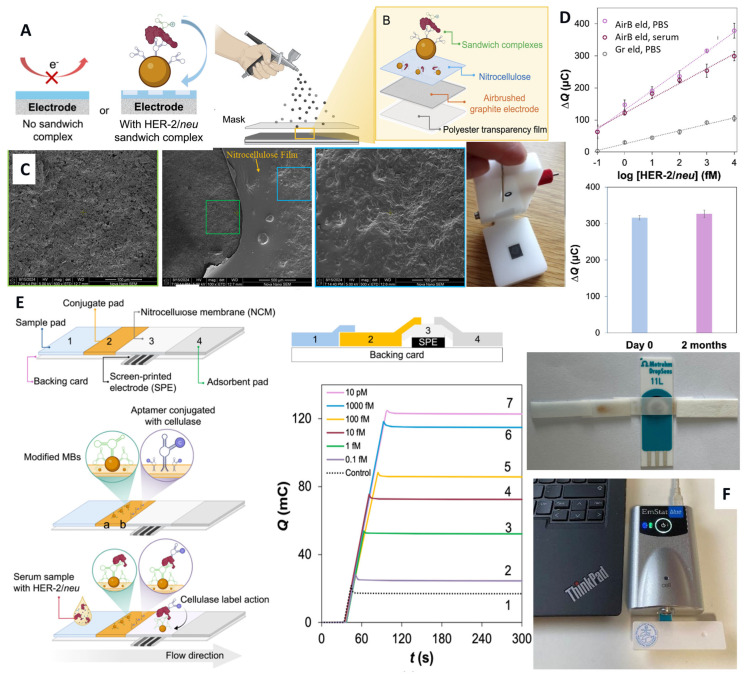
(**A**) Schematic of the electrochemical cellulase-linked aptamer assay (e-ELASA) on magnetic beads for HER-2/neu detection on nitrocellulose-covered electrodes and (**B**) the preparation of the airbrushed graphite electrodes; (**C**) top-down SEM images of the (left panel) bare airbrushed electrode and (right panel) nitrocellulose-modified electrode, areas enlarged are marked in the central image (magnification: center-100× and side panels-500×) and the image of the airbrushed electrode assembled in the e-cell. (**D**) Response of the airbrushed electrodes (AirB eld) to HER-2/*neu* in PBS and serum and that of manually polished graphite electrodes (Gr eld) in PBS and electrode storage stability [78]. (**E**) Image and schematic of the electrochemical lateral flow test (e-LFT) for HER-2/*neu* using the cellulase-linked aptamer sandwich strategy and screen-printed carbon electrodes: top view and side view; a and b denote points at which aptamer modified-magnetic beads (MBs) and a cellulase-conjugated aptamer are drop-casted, respectively. (**F**) A fully assembled e-LFT device in a plastic casing connected to the portable PalmSens potentiostat and the chronocoulometric responses of the e-LFT strips to HER-2/*neu* [79]. Copyright (2025) Elsevier and (2026) Americal Chemical Society, reprinted with permission.

Sandwich e-ELASA and e-ELISA further enhanced both the LOD and overall sensitivity for HER-2/*neu* detection. Using a nanoparticle-based silver enhancement step, HER-2/*neu* could be quantified down to 1.4 fM in 4% serum within 1 h 10 min [75]. HER-2/*neu* was captured by antibodies immobilized on the AuNP-modified electrode, while a reporter aptamer carrying hydrazine-modified AuNP tags was subsequently introduced. The AuNPs were silver-enhanced and finally quantified by voltametric stripping. Another, non-conventional e-ELASA employed a hydrolase label, offering a signal-generation mechanism as an alternative to the one involving traditional redox enzymes [76]. Cellulase-linked e-ELASA, performed on magnetic beads and employing two identical dimerized aptamers as both capture and reporter elements (Figure 6A), enabled detection of HER-2/*neu* ECD in the serum of healthy individuals within 1.2 h [55]. The assay exploited changes in the capacitive properties of highly porous spectroscopic graphite electrodes that exhibited capacitive current densities of 0.4–2 mA cm^−2^ [76]. These currents decrease by 90–95% after the electrodes were coated with a nitrocellulose film. Partial digestion of the nitrocellulose layer by the cellulase-labeled aptamer–protein–aptamer sandwich, formed on magnetic beads and applied to the electrode surface, resulted in a significant increase in the capacitive currents, thereby enabling protein detection at concentrations as low as 0.1 fM. Given the relatively high serum concentration of HER-2/*neu* (ranging from sub-picomolar levels to ~10 pM in healthy individuals [55], and hundreds of picomolar in cancer patients), such ultralow protein detection enables accurate analysis of diluted serum samples. This reduces or even eliminates serum interference and, more importantly, prevents sensor oversaturation by the target protein at high concentrations, thereby enabling reliable analysis of cancer patients’ samples. In perspective, low-LOD systems may be extended to the analysis of other biofluids, such as saliva and urine, where biomarker concentrations are typically much lower than in serum. Similar low-LOD strategies are applicable to other cancer protein biomarkers, which will be discussed further.

The cellulase-linked e-ELASA platform was further advanced to electrochemical ELNASA (nanobody–aptamer–sorbent assay). The capture antibody was replaced by either a single nanobody or a combinatorial pair of nanobodies, 2Rs15d and 2Rb17c, which bound two distinct and non-overlapping domains of the HER-2/*neu* ECD [77]. Owing to their small size, nanobodies enabled a higher surface loading density on magnetic beads. The use of a single nanobody receptor reduced the immunoassay sensitivity by 26–35%, whereas the combination of two nanobodies as a dual-receptor system restored e-ELNASA sensitivity through the avidity effects, rendering the binding performance comparable to that of conventional antibodies. Moreover, in a clinical study involving 30 healthy volunteers, ELNASA outperformed optical ELISA in the accuracy of serum HER-2/*neu* measurements. Due to the lower production costs of both nanobodies and aptamers, e-ELASA and ELNASA, estimated to be 200–300 times cheaper than conventional ELISA, are better suited for routine POC analysis, particularly in low- and middle-income settings with limited resources. This was further demonstrated using disposable, high-capacitance graphite electrodes (specific capacitance ranging from 3.61 to 8.88 mF cm^−2^) airbrushed onto plastic supports (Figure 6B–D). These electrodes were fabricated from water-based inks composed of graphite microparticles as the conductive component, chitosan as a water-soluble polymer matrix, and glycerol as an emulsifying agent, and showed improved sensitivity of serum HER-2/*neu* detection compared to Gr electrodes [78]. Recent adaptation of the assay to a lateral flow format reduced the total assay time (from addition of a 150 µL serum sample to signal readout) to just 5 min, without compromising sensitivity of the assay (Figure 6E,F) [79].

Further advancement of e-ELASA on magnetic beads was achieved through the use of novel O_2_-dependent electrocatalytic labels, such as covalent hemin–guanine quadruplex (G4) complexes [80]. This DNAzyme-linked e-ELA/ISA enabled detection of serum HER-2/*neu* at 10 fM (aptamer–aptamer sandwich) and 1 fM (aptamer–antibody sandwich) within 1 h. The biosensor performance relied solely on ambient O_2_ present in solution and did not require adding any extra reagents. The high efficiency of O_2_ reduction electrocatalysed by this DNAzyme was attributed to the large surface area of the graphite electrode employed.

In conclusion, the overview of recent advances indicates that most of the discussed HER-2/*neu* biosensors can be directly applied to serum analysis (Table 2).

### 5.2. PSA

The test for serum prostate-specific antigen (PSA) is one of a few approved by FDA for prostate cancer diagnosis [81]. PSA is a serine protease produced by the prostate gland, whose enzymatically active form plays a role in the liquefaction of the seminal ejaculate. The concentration of PSA in the bloodstream, arising from leakage through the prostate epithelium, is typically below 4.9 ng mL^−1^ in healthy individuals, depending on age and prostate size. In contrast, PSA levels can readily exceed 20 ng mL^−1^ in the blood of high-risk prostate cancer patients. Serum-circulating PSA is, therefore, widely used as a clinical biomarker for the diagnosis and monitoring of prostate cancer. However, PSA is not specific to prostate cancer and is also elevated in benign prostatic hyperplasia and prostatitis, which leads to false-positive prostate cancer results [82,83] and, thus, overdiagnosis and overtreatment of a low-risk disease [84]. Recent clinical trials have questioned the utility of PSA as a standalone diagnostic biomarker; nevertheless, PSA testing is still widely used for population-based prostate cancer screening [85,86], and numerous electrochemical biosensor strategies for PSA analysis have been reported and extensively reviewed [9,87,88,89,90,91].

**Figure 7:**
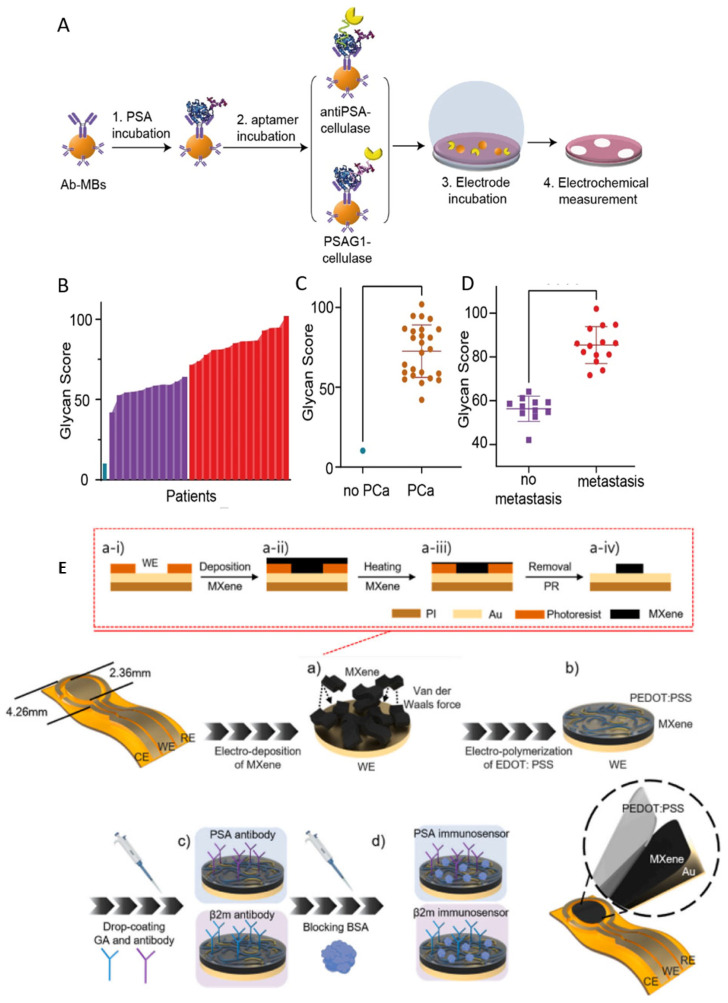
(**A**) Schematic representation of electrochemical cellulase-linked immuno-aptamer assay (e-ELI/ASA) on magnetic beads for total PSA (t-PSA) and glycosylated PSA (g-PSA): sandwich assembly and electrochemical detection of t-PSA with a PSA aptamer and a g-PSA aptamer, both conjugated to cellulase. (**B**) Waterfall distribution of the Glycan Score values in the serum samples of prostate cancer (PCa) patients: (blue) non PCa, (purple) non metastatic PCa, and (red) metastatic PCa. (**C**,**D**) Correlation between the Glycan Score and (**C**) the diagnosis of (brown) PCa patients, in (blue) no PCa patient; and (**D**) the cancer state, (red) metastatic and (purple) primary prostate cancer [98]. Copyright (2025) ACS, reprinted with permission. (**E**) Schematic representation of the stepwise (steps **a**–**d**) preparation of the biosensor for PSA detection based on MX/PP proposed in [102]. Subfigures ((**a-i**)–(**a-iv**)) illustrate the steps involved in MXene preparation. Copyright (2025) Elsevier, reprinted with permission.

Despite multiple electrochemical PSA assays reported, they have not significantly improved the accuracy of prostate cancer diagnostics. Recently, the selection of a novel aptamer with specificity for both PSA glycans and its surface peptide regions [92] allowed a more precise stratification of prostate cancer patients versus healthy individuals, based on analysis of post-translational glycosylation of PSA. In prostate cells, PSA is produced as a glycoprotein with N- and O-glycosylation sites at Asn61 and Thr125, respectively. In healthy individuals, serum PSA features bi-antennary glycans with core fucose, N-acetylgalactosamine, and terminal sialic acids [93]. Aberrant post-translational glycosylation of PSA, associated with cancer development and progression [94,95], produces a mixture of bi- and tri-antennary glycans with decreased sialylation and elevated fucose and N-acetylgalactosamine content, accompanied by increased core fucosylation [96]. In a trial involving 12 participants, an e-ELISA assay performed on gold electrodes, using a horseradish peroxidase label and a novel reporter aptamer recognizing PSA glycans, successfully discriminated prostate cancer patients from individuals without cancer [97]. The extent of serum PSA glycosylation was quantified by evaluating a so-called PSA Glycan Score, defined as the ratio of glycosylated PSA to total PSA in serum. Furthermore, in a cellulase-linked dual-functional aptamer–antibody assay, the PSA Glycan Score achieved 100% accuracy in determining prostate cancer status in a cohort of 30 patients, correctly stratifying individuals with primary and metastatic prostate cancer [98] (Figure 7). It was suggested that the extent of sensor-detectable glycosylation of PSA circulating in the serum of cancer patients was likely associated with cancer-related disruption of PSA complexes with serum proteins [98]. Accordingly, serum PSA levels alone correctly classified only 53% of prostate cancer patients and failed to reliably inform disease status. Thus, aberrant glycosylation of PSA linked to cancer development and progression can now be suggested as a key target in prostate cancer research [99], and the PSA Glycan Score emerges as a more accurate liquid biopsy biomarker for the classification and prognosis of prostate cancer. Consequently, the Glycan Score-based liquid biopsy test holds strong potential for precise prostate cancer diagnosis and staging, supporting the advancement of mass-screening programs and improving monitoring of advanced prostate cancer treatment.

Nevertheless, in recent years most analytical efforts have shifted toward ultrasensitive quantification of total PSA in serum, while detailed analyses of PSA glycosylation patterns and derived glycoscores have largely remained underexplored despite their apparent clinical relevance (Table 3). Activated carbon from marigold flowers (MG) combined with graphene quantum dots (GQDs) was used to produce a highly porous, conductive scaffold to support poly(thionine), which acted as an intrinsic redox mediator [100]. Anti-PSA antibody was coupled to modified screen-printed electrodes (SPEs) via glutaraldehyde, and PSA binding modulated the poly(thionine) (PTH)-mediated electron transfer (ET). Due to the large electroactive area and fast ET within the MG/GQD/PTH film, this label-free immunosensor reached a LOD of 0.005 ng mL^−1^ (5 pg mL^−1^) and performed well in human serum. Other platforms exploited MXene-based or mesoporous nanoarchitectures with built-in electrocatalysts. A sandwich-type PSA immunosensor was constructed based on polyaniline-loaded Ti_3_C_2_ MXene quantum dots and AuNPs, forming the conductive, redox-active backbone; with V_2_C MXene QDs–AuNP conjugates serving as signal tags [101]. MXene QDs provided a high density of edge sites and supposedly fast ET pathways, while PANI and AuNPs further boosted film conductivity. This MXene nanozyme–like interface yields a remarkable LOD of 0.61 fg mL^−1^ detected voltametrically. Another MXenes-based platform was suggested for a dual urinary immunosensor [102]. A gold electrode was coated with MXene, and PEDOT:PSS was electropolymerized on its surface (MX/PP), producing a highly conductive, high-surface-area interface for co-immobilization of PSA- and β_2_-microglobulin-specific antibodies. The MX/PP layer acted as an efficient electrocatalytic/conductive transducer converting protein binding into charge-transfer resistance changes detectable by EIS, enabling a LOD of 4.96 × 10^−5^ ng mL^−1^ for PSA detection in urine, with clear prostate cancer vs. benign condition discrimination (Figure 7). In a related but structurally more complex system, mesoporous carbon nanospheres loaded with ferrocene (Fc) carboxylic acid and hemoglobin (Hb) (HMCNs-FCA-Hb) were used [103]. FCA acted as a built-in redox reporter released under alkaline conditions of PSA detection, while Hb’s peroxidase-like activity enabled indirect sarcosine readout. Combination of the mesoporous carbon reservoir and Fc/Hb co-catalysis allowed PSA detection down to 0.11 pg mL^−1^ in a compact, micro-workstation-integrated device.

The next step to developing highly miniaturized devices with electronic readouts was reported in [104], where authors assembled a peptide-based PSA sensor on single-walled carbon nanotubes (SWCNT) bridging a gold fork electrode. The SWCNT network functioned as a highly conductive, high-aspect-ratio support, while PSA-specific peptides provided recognition. PSA binding to the peptide affected the electronic properties of the SWCNT network, which was recorded in the absence of any external redox species. This FET-like configuration achieved a surprisingly low LOD on the order of 10^−13^ µg µL^−1^ (~0.1 fg mL^−1^), rivaling and outperforming many redox-based assays. However, the clinical significance of such extraordinarily low LOD for prostate cancer diagnostics remains unclear, as even when serum samples are diluted to mitigate electrode biofouling, the clinically targeted PSA concentrations in the ng mL^−1^ range remain far above the demonstrated LOD.

### 5.3. EGFR

Epidermal growth factor receptor (EGFR) is a tyrosine kinase belonging to the transmembrane receptor ErbB family; upon binding ligands such as EGF or TGF-α, EGFR dimerizes and auto phosphorylates its intracellular kinase domain, triggering downstream signaling pathways (RAS–MAPK, PI3K–Akt, JAK–STAT) that regulate cell proliferation, survival, migration, and differentiation [105,106]. In adult tissues, EGFR is expressed at from relatively low to moderate levels, mainly in epithelia, where it contributes to tissue homeostasis and wound repair; in cancer, however, it is frequently overexpressed or mutated and functions as a key oncogenic driver [107]. High expression of EGFR and/or its activating mutations are characteristic of non-small cell lung cancer, colorectal cancer, head and neck squamous cell carcinoma, glioblastoma, and subsets of breast, bladder and ovarian cancers, where EGFR status guides prognosis and the use of targeted therapies (e.g., TKIs or anti-EGFR antibodies) [108,109,110,111]. The combination of strong mechanistic involvement in tumor development and recognized clinical relevance of EGFR made it a desired biomarker for cancer detection, and in recent years, a range of highly sensitive electrochemical platforms have been developed to detect either circulating EGFR or EGFR mutations.

Several systems exploited highly conductive, redox-active interfaces for EGFR detection (Table 4). An immunosensor used SiO_2_-nanosphere-modified gold electrodes for the construction of amperometric silver stripping biosensor for EGFR [112]. The nanospheres dramatically increased the surface area and surface loading with capture antibodies, while biotin-labeled aptamers and alkaline phosphatase label were used to generate an electroactive product that drove silver deposition on the electrode surface. The Ag-deposition electrocatalytically amplified the EGFR-associated signal, enabling highly sensitive (down to 0.06 ng mL^−1^) yet quite expensive, due to the reagents used, detection of EGFR. The authors of [113] instead designed a low-cost electrochemical paper-based device (ePAD) for detecting uterine cancer, using silver nanowires as a conductive working electrode, an inexpensive EGFR aptamer as a recognition element, and [Fe(CN)_6_]^3−^/^4−^ as a redox indicator. The nanowire network provided high surface area and fast ET at a disposable paper substrate. ET between the Ag nanowires and [Fe(CN)_6_]^3−^/^4−^ was hindered after EGFR binding, and the suppression of the voltametric signal yielded a LOD of 0.01 ng mL^−1^.

**Figure 8:**
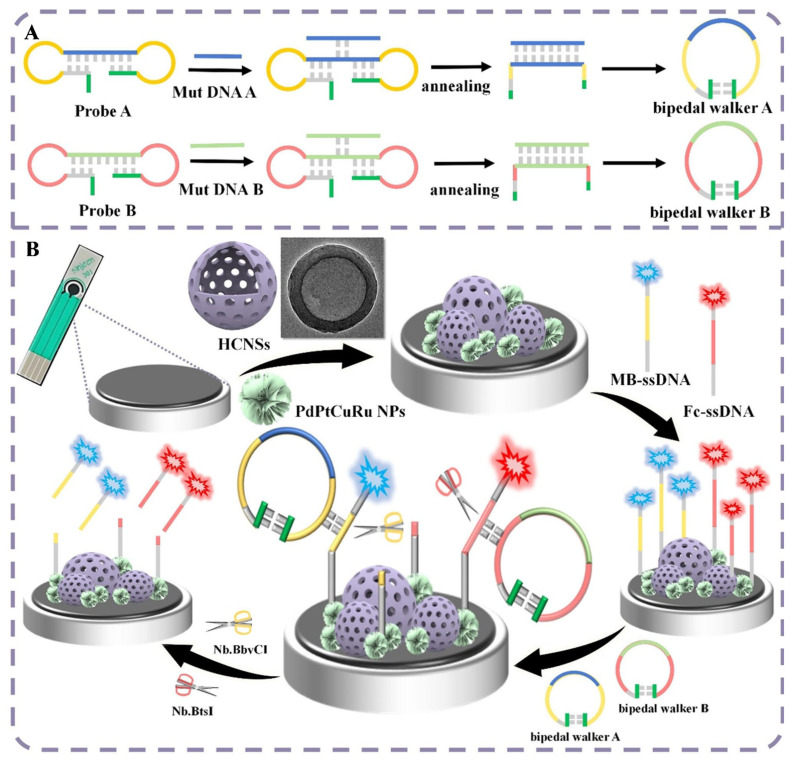
Method of detecting mutations in exon 19 of EGFR: E746_A750del (in (**A**): Mut DNA A, marked in blue) and L747_S752delinsS (in (**A**): Mut DNA B, marked in light green) using the circular bipedal DNA walkers A and B [114]. In (**B**): blue and red labels stand for methylene blue (MB) and ferrocene (Fc) labelling the corresponding ssDNA probes. Copyright (2025) Springer, reprinted with permission.

Complementing these EGFR-targeting immunosensors, EGFR exon 19 mutations were targeted instead of the protein itself, integrating circular bipedal DNA walkers with a 3D hollow carbon nanosphere–tetrametallic nanoparticle (3D HCNs–TT NPs) platform (Figure 8) [114]. The 3D HCNs–TT composite provided a highly conductive, high-surface-area electrode with abundant active sites, markedly enhancing ET and supporting dense loading of DNA probes. Signal amplification was achieved through the autonomous motion of circular bipedal DNA walkers on the nanostructured surface, which repeatedly cleave or release electroactive reporters in response to two specific EGFR mutant sequences (E746_A750del and L747_S752delinsS). This combination of catalytic DNA walking and electrocatalytically efficient 3D HCNs–TT NPs allowed the detection of ultrasensitive mutations, with LODs down to 3.4 fM and 2 fM for the two mutations, respectively, in cells and diluted serum. Taken together, these examples illustrate how EGFR, whether as an overexpressed protein or as oncogenic DNA variants, can be probed through Ag-deposition chemistry, silver nanowire/[Fe(CN)_6_]^3−/4−^ redox chemistry, or using 3D carbon–metal nanocomposites, consistently pushing EGFR detection into the low pg mL^−1^ or femtomolar regime while keeping formats compatible with flexible, disposable, or clinically translatable devices.

### 5.4. α-Fetoprotein

Alpha-fetoprotein (AFP) is a 70 kDa oncofetal glycoprotein that is overexpressed during embryonic development by the fetal liver, yolk sac, and gastrointestinal tract but drops to very low concentrations in healthy adults [115,116,117]. Pathologically, its re-expression and strong elevation in serum are characteristically associated with hepatocellular carcinoma (HCC) and non-seminomatous germ cell tumors (especially yolk sac tumors and mixed germ cell tumors), where AFP is routinely used for diagnosis, risk stratification, and monitoring of treatment response or recurrence; it is FDA-approved as a serum biomarker for HCC and testicular germ cell cancer [118,119]. Raised AFP levels have also been reported in subsets of gastric, pancreatic, and other gastrointestinal cancers, and its modest elevation can occur in chronic liver diseases such as viral hepatitis and cirrhosis, which compromises its specificity but underscores its tight link to hepatocellular injury and malignant transformation [120].

**Figure 9:**
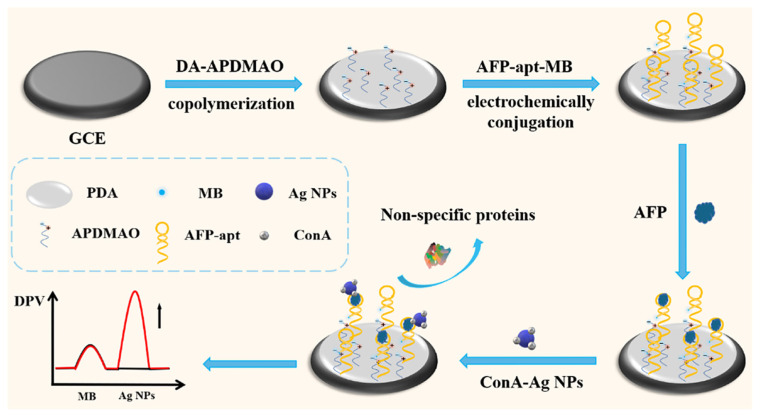
Schematic diagram of the construction process of the antifouling electrochemical biosensor for AFP detection based on AgNPs and MB catalytic activity [122]. Copyright (2024) ACS Publications, reprinted with permission.

High serum AFP generally correlates with tumor burden, vascular invasion, and poorer prognosis in HCC, and because clinically relevant changes often occur at low ng mL^−1^ or even sub-ng mL^−1^ levels in a highly fouling matrix (like serum) [121], there is strong motivation to develop biosensing platforms that combine ultra-high sensitivity with robust antifouling resistance (Table 5). To address these challenges, an elegant antifouling electrochemical aptasensor for AFP was developed based on biomimetic zwitterionic 3-aminopropyldimethylamine oxide (APDMAO) co-polymerized with dopamine to form a thin, hydrated film on the electrode surface [122]. The N^+^–O^−^ zwitterion built a dense hydration shell that effectively suppressed nonspecific adsorption, while amine groups provided anchoring sites for thiolated, methylene-blue (MB)-labeled AFP aptamers and concanavalin A–silver nanoparticle (ConA–AgNP) signal probes. Using a ratiometric strategy, in which MB acted as an internal redox standard and the AgNP nanozyme contributed an amplified electrocatalytic response, the sensor minimized background and instrumental drift and achieved highly sensitive AFP detection in serum with a LOD of 3.41 fg mL^−1^ over a wide dynamic range. This approach highlights how zwitterionic antifouling chemistry, built-in redox labels, and Ag-based electrocatalysis can be synergistically combined for clinically relevant liver cancer biomarker monitoring (Figure 9).

Built on this zwitterionic MB/Ag-assisted platform, a number of AFP sensors exploiting nanostructured electrocatalysts and aptamer architectures have been further suggested to combine high sensitivity with clinically useful formats. A signal-on electrochemical aptasensor was designed, in which an AuNPs–MXene composite was drop-cast on portable microelectrodes and used as a highly conductive, high-surface-area support for MB-labeled AFP aptamers [123]. Upon AFP binding, target-induced folding brought the MB label closer to the AuNPs–MXene surface, enhancing ET and generating a “signal-on” response rather than more common signal-off change. This simple conformational beacon, coupled to the electrocatalytic properties of the AuNPs–MXene scaffold, enabled selective AFP quantitation in serum with a LOD of 0.05 ng mL^−1^, while maintaining good stability and reproducibility in real-world samples.

Moving beyond single-analyte readouts, a dual-mode aptasensor, which reads signals from two distinct electrocatalytic nanoprobes labeling two protein biomarkers, correspondingly, was designed for simultaneous determination of AFP and Golgi protein 73 (GP73) [124]. In the GP73 channel, an rGO–ferrocene (Fc)–polyaniline nanocomposite provided a conductive backbone and a robust Fc signal that was enhanced upon GP73 binding via aptamer/cDNA dissociation. For AFP detection, nitrogen-doped rGO–Cu_2_O nanostructures were used, whose redox response was suppressed by the aptamer–AFP complex formation and associated dielectric changes. This bidirectional signal design allowed independent, yet parallel, quantification of both biomarkers in a single platform, with a LOD of 1.77 pg mL^−1^ for AFP, illustrating how graphene-based redox polymers and Cu_2_O nano-electrocatalysts can be combined for multiplexed HCC diagnostics. In constrast, multi-functionalization of graphene was suggested solely for AFP immunosensing [125]. A quite complicated sensor platform included a TB–Au–Fe_3_O_4_–rGO nanocomposite incorporating a Ru-like organic redox dye (toluidine blue, TB), Au NPs, and magnetic Fe_3_O_4_ particles assembled within the reduced graphene scaffold. The modified electrode exhibited a reduced charge-transfer resistance of ~ 43 Ω, consistent with good ET properties provided by rGO and Au and high catalytic activity toward the TB redox transformation. Direct immunosensor constructed by immobilizing anti-AFP antibodies on the modified electrodes detected down to 0.03 ng mL^−1^ AFP with minimal interference from abundant serum proteins and a response time of ~35 s; sensor results agreed well with those of ELISA.

Semiconductor-based nanocomposites have been employed to improve the sensitivity of AFP detection. An aptasensor was designed based on water-soluble CdTe/CdSe/polyaniline nanocomposite that acted simultaneously as a high-gain electroactive film and an electrocatalyst for K_3_Fe(CN)_6_ oxidation [126]. The multiple-sensitized CdTe/CdSe quantum-dot structure promoted rapid charge transfer and suppressed electron–hole recombination, while the PANI matrix ensured good conductivity and abundant carboxyl groups for covalent immobilization of the AFP-specific aptamers. In the presence of AFP, formation of the aptamer–AFP layer created steric hindrance and inhibited ET reaction of ferricyanide, leading to AFP concentration-dependent decrease in the detectable current. Synergistic QD–PANI electrocatalysis and catalytic oxidation of [Fe(CN)_6_]^3−^, allowed 1.0 pg mL^−1^ detection of AFP in serum, with good reproducibility and specificity. Electrochemiluminescent (ECL) approaches were also explored for ultrasensitive detection of AFP, by combining graphitic carbon nitride (g-C_3_N_4_) and polypyrrole (PPy) into a heterojunction emitter layer, integrating both molecular imprinting and aptamer recognition for AFP [127]. The g-C_3_N_4_/PPy heterostructures improved charge separation and facilitated participation of more electrons in ECL, enhancing signal stability and intensity, while the dual-recognition architecture (MIP + aptamer) strengthened AFP binding at the electrode. Under optimized conditions, the ECL sensor enabled an extremely low LOD of 10^−11^ µg mL^−1^, emphasizing how coupling organic semiconductor heterojunctions with complementary molecular-recognition motifs can drive AFP detection to ultralow levels.

### 5.5. Osteopontin

Osteopontin (OPN), also known as secreted phosphoprotein-1 (SPP1), is a phosphorylated, highly glycosylated matricellular protein secreted into the extracellular space and bodily fluids, where it binds to integrins (e.g., αvβ3, αvβ5) and CD44 variants to modulate cell adhesion, migration, survival, and immune responses [128,129]. Under physiological conditions, it is involved in bone remodeling, wound healing and inflammation, but in cancer, it is frequently overexpressed and abundantly secreted by tumor cells, stromal cells, and infiltrating immune cells [130]. Elevated OPN expression has been reported in a wide range of malignancies, including breast, lung, colorectal, gastric, pancreatic, hepatocellular, prostate, and ovarian cancers, as well as gliomas and several hematological tumors, where it is closely linked to invasion, epithelial–mesenchymal transition, angiogenesis, immune evasion, and the formation of distant metastases—particularly in bone [131,132]. High OPN levels in tumor tissue and in circulation (serum, plasma and, in some settings, urine) are often associated with advanced stage and poor prognosis, and dynamic changes can reflect treatment response or disease progression. This combination of OPN’s functional role in tumor biology, broad overexpression across cancer types, and ready accessibility as a soluble biomarker has driven growing interest in OPN as a diagnostic and prognostic indicator [131].

Building on this biological relevance, several electrochemical strategies have been explored for OPN detection in serum, designed to compensate for its non-electroactive nature (Table 6). To address the latter, an internal redox probe was embedded within a molecularly imprinted polymer (MIP) film on an acupuncture needle microelectrode: MB and dopamine were electropolymerized around OPN templates to yield a surface MIP with built-in poly(methylene blue) (pMB) confined in the imprinted nanocavities [133]. Upon rebinding of OPN, mass transport and charge transfer to the pMB domains were hindered, causing an OPN concentration-dependent decrease in the current. This smart “self-reporting” design enabled direct voltametric analysis of OPN with a LOD of 3 pg mL^−1^, high selectivity against coexisting glycoproteins, and good sensor stability. A reusable electrochemical immunosensor for continuous bioprocess monitoring with K_3_[Fe(CN)_6_] as a redox indicator was suggested to track secreted OPN from differentiating human mesenchymal stem cells in a microfluidic bioreactor [134]. Efforts were focused on designing stable, label-free immunosensor, constructed by the immobilization of biotinylated OPN-specific antibodies on 11-mercaptoundecanoic acid SAM conjugated to aminated streptavidin, and enabling detection of trace OPN in complex culture media over weeks. The reported platform showed a low LOD of 0.171 pg mL^−1^, high sensitivity, and excellent selectivity in protein-rich environments. It was used to follow dynamic OPN secretion in 2D and 3D osteogenic cultures for more than a month, supporting long-term OPN readout.

A conceptually different amplification approach for sensitive OPN detection used the coupling of RNA aptasensing on magnetic α-Fe_2_O_3_/Fe_3_O_4_ nanosheets with the collateral cleavage activity of CRISPR/Cas13a [135]. The α-Fe_2_O_3_/Fe_3_O_4_ nanocomposite served as a conductive and magnetically addressable matrix for assembling the sensing aptamer layer, while OPN binding to the aptamer displaced an activator strand that subsequently triggered the Cas13a activity. Once activated, Cas13a indiscriminately cleaved the reporter nucleic acids tethered to the electrode, altering the interfacial resistance/current as a function of the OPN concentration. This CRISPR-driven enzyme-amplified electrocatalytic system yielded a LOD of 0.33 pg mL^−1^ and showed good selectivity, reproducibility, and stability. In this approach, nucleic acid biocatalysis was harnessed to push OPN detection to sub-picogram per mL levels.

**Figure 10:**
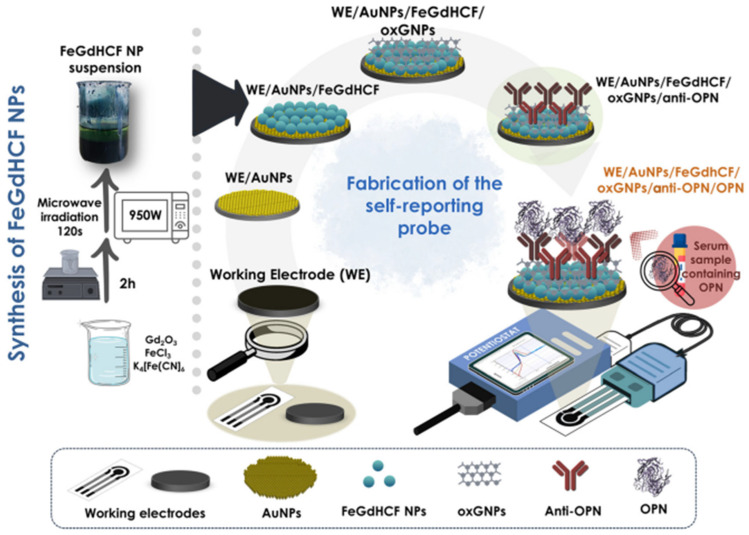
Schematic representation of the redox nanoparticle synthesis and step-by-step procedure involved in the fabrication of the electroactive immunosensor probe based on electrodeposited gold and the Prussian-blue-type redox nanozyme [136]. Copyright (2025) Royal Society of Chemistry, reprinted with permission.

Focusing on the mediator-free POC operation of the miniaturized device, electroactive antibody-modified surface was engineered, in which redox functionalities resided on the electrode, eliminating the need for soluble redox indicators (Figure 10) [136]. This OPN immunosensor integrated gold electrodeposited on SPEs, a bimetallic Prussian-blue-type redox nanozyme FeGdHCF, and oxidized graphene nanoplatelets as a nanocomposite transducer that supported antibody immobilization. Binding of OPN modulated the redox response from the nanozyme and built-in the sensing layer in a buffer and serum, enabling label-free voltametric detection of OPN at down to 0.437 pg mL^−1^ levels; the immunosensor showed stable performance over six weeks and was suggested to be a promising tool for sensitive, point-of-care-compatible OPN detection for osteosarcoma monitoring in serum.

### 5.6. Mucins

Mucins are a family of high-molecular-weight, heavily O-glycosylated transmembrane or secreted glycoproteins that coat epithelial surfaces and are frequently dysregulated in malignancy [137]. Among them, MUC1 is a prototypical tumor-associated mucin, which, although normally restricted to the apical membrane of epithelial cells, becomes overexpressed (the cancer cut-off level of MUC1 is 3.96 pM) [138] and aberrantly glycosylated in a broad spectrum of carcinomas, most notably breast, ovarian, pancreatic, and lung cancer [139]. In these settings, MUC1 overexpression and altered glycosylation contribute to loss of polarity, increased motility, resistance to apoptosis, and immune evasion, and have been associated with higher tumor grade, metastatic potential, and poor clinical outcome [140,141]. Tumor-associated processing of MUC1, including proteolytic cleavage, ectodomain shedding and release in soluble or vesicle-associated forms, gives rise to circulating epitopes such as CA 15-3 [141], the most widely used serum marker for breast cancer monitoring and recurrence assessment, where dynamic changes in CA 15-3 levels can reflect treatment response or emerging relapse, despite well-known limitations for early-stage screening [141,142,143]. In parallel, MUC16, detected clinically as CA 125, is overexpressed and shed in ovarian and other gynecological cancers, where it plays a role in peritoneal dissemination, immune modulation, and chemoresistance [144], and remains a cornerstone serum marker for disease staging, follow-up, and evaluation of residual or recurrent disease. Beyond their individual clinical uses, MUC1, CA 15-3, and MUC16/CA 125 exemplify how tissue overexpression and systemic shedding of tumor-associated mucins can be exploited in complementary ways, via histopathological assessment in solid lesions and longitudinal quantification in blood, to capture tumor burden and biological aggressiveness [145]. Together, MUC1, CA 15-3, and CA 125, thus, define a clinically established axis of mucin-type markers spanning tumor tissue and circulation across several major epithelial cancers, which explains why they dominate current efforts in biosensor/bioelectrocatalysis development [145]. Several electrochemical approaches have been explored to develop sensing platforms for mucins and derivatives in recent years. 

#### 5.6.1. MUC1

Diverse assay formats for MUC1 detection that go beyond conventional immunoassays have been proposed, incorporating biocatalytic and electrocatalytic amplification schemes to address the low abundance, high heterogeneity, and complex matrix effects typical of circulating mucins. A label-free MUC1 aptasensor was proposed based on a nanohybrid electrocatalytic interface composed of goldnanoparticle-decorated three-dimensional Cd–Co oxide nanostructures (3D-CdCo-ONSs@AuNPs) [146]. In this architecture, the 3D-CdCo-ONSs provided a high-surface-area conductive scaffold for dense AuNP deposition, whereas deposited gold domains enable efficient immobilization of thiolated MUC1 aptamers via Au–S bonds. MUC1 binding decreased the voltametric signal at −0.8 V, without the need for additional enzymatic or other electrocatalytic labels, which enabled ultralow detection of MUC1 with a LOD of 0.96 pg mL^−1^ across a broad, eight orders of magnitude linear range. The aptasensor showed good reproducibility and stability (16% signal loss after 5 days of storage) and was suitable for MUC1 quantification in MUC1-spiked 10% diluted serum and in clinical serum samples, yet the results of the latter analysis were not benchmarked against alternative assays. In a more complex conceptually related design, a dual “signal-on/off” electrochemical MUC1 aptasensor was developed combining nucleic acid-based CRISPR-Cas12a biotechnology and redox-active interface incorporating electrocatalytic nanohybrids [147]. The addition of MUC1 triggered the catalytic hairpin assembly (CHA) coupled to a DNA-tetrahedron-mediated nonlinear hybridization chain reaction (HCR), greatly enriching magnetic nanoparticles with MB molecules and turning the MB signal “on”. In parallel, isothermal amplification products activated CRISPR–Cas12a, which cleaved ssDNA at the electrode surface, thus, decreasing the loading of a ferrocene-containing MgAl-LDH@Fc-AuFe-MIL-101 hybrid on the electrode surface, switching the Fc signal “off”. Two redox signals (MB and Fc) were modulated in opposite directions and jointly calibrated, with a LOD of 5.97 fg mL^−1^ (detected using MB signals) and 0.43 fg mL^−1^ (detected using Fc signals), enabling voltametric MUC1 determination in clinical serum samples.

In addition to purely voltametric formats, MUC1 has also been targeted by electrocatalytically enhanced ECL. The inherently low and unstable response of conventional luminol-based ECL was combated in the Au/ZnCuS double-enhanced luminol aptasensor supported by g-C_3_N_4_ [148]. In this approach, g-C_3_N_4_ provided a high-surface-area substrate for loading large amounts of luminol, while AuNPs and ZnCuS nanostructures acted as co-catalysts for H_2_O_2_ decomposition. AuNPs promoted generation of reactive oxygen intermediates (•OH, O_2_•^−^), and ZnCuS both anchored the MUC1 aptamer and further catalyzed H_2_O_2_ breakdown, yielding a dual “double wing” signal amplification. Under optimized conditions, the sensor detected down to 5 × 10^−5^ ng mL^−1^ MUC1, and showed good stability, selectivity, and reproducibility [148]. Extending these electrocatalytic concepts from single- to multi-biomarker analysis, an electrochemical dual immunosensor was developed for simultaneous detection of HER2 and MUC1, aiming to improve early breast cancer diagnosis through multi-biomarker readout [149]. The platform combined Ti_3_C_2_T_X_ MXene loaded with Ru/PdO nanostructures (Ru/PdO@MXene) with a poly(aniline-co-pyrrole) copolymer film, where the MXene–Ru/PdO hybrid provided a highly conductive and catalytically active stable support for impedimetric analysis while the copolymer layer facilitated antibody immobilization. The dual sensor exhibited an ultralow LOD of 0.28 fg mL^−1^, for MUC1, and high sensitivity within short (ca. 20 min) assay times, and it was used for MUC1 analysis in MUC1-spiked serum.

#### 5.6.2. CA 15-3

**Figure 11:**
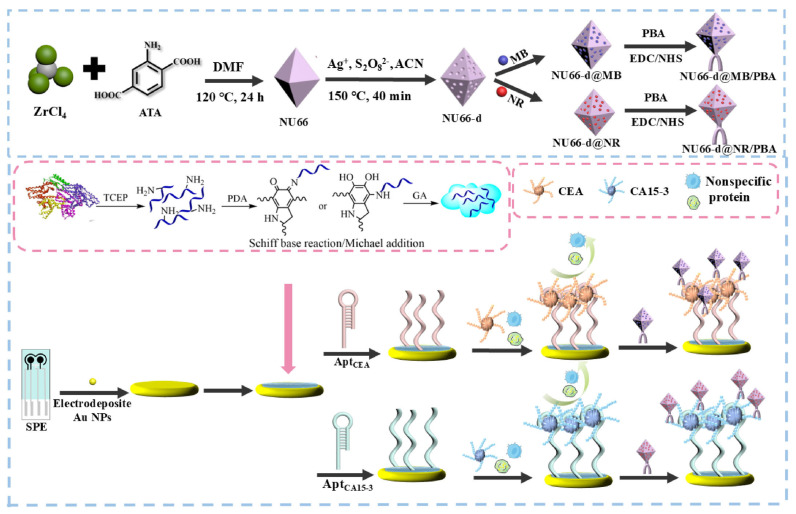
Schematic diagram of the preparation antifouling electrochemical sensor for the detection of CEA and CA15-3 based on UiO-66-d MOF [151]. Copyright (2026) Elsevier, reprinted with permission.

Recent achievements in CA 15-3 detection include an electrochemical sensor constructed using CA15-3-specific MIP nanoparticles (nanoMIPs) integrated in a nanostructured electrode and ferricyanide as a redox indicator [150]. The nanoMIPs were produced by solid-phase synthesis and subsequently anchored to carbon SPEs modified with multi-walled carbon nanotubes (MWCNT) and AuNPs, creating a hybrid interface with increased electroactive surface area and facilitated ET, thereby enhancing voltametric readouts. Under optimized conditions, the sensor exhibited a LOD of 0.14 U mL^−1^, comfortably spanning the clinical reference threshold of 30 U mL^−1^ and was used for analysis of spiked serum samples (samples’ pH adjusted to pH 4.5), yet no real samples analysis was eventually performed, and nanoNIP (a not imprinted system) system showed relatively high interference. A portable dual-channel electrochemical aptasensor for CA 15-3 was developed for simultaneous determination of carcinoembryonic antigen (CEA) and CA15-3, further illustrating how CA15-3 can be integrated into multiplexed POC formats (Figure 11) [151]. A glutaraldehyde-crosslinked denatured BSA/polydopamine coating on SPEs provided an antifouling interface, while etched UiO-66-d MOF particles were used as high-capacity hosts for methylene blue and neutral red redox probes loading, which were then functionalized with phenylboronic acid (PBA) to generate two distinct redox tags. Aptamer- and PBA-based recognition of CEA and CA15-3 was performed via the formation of sandwich architectures on a dual-channel SPE so that each biomarker was reported by a separate MOF-based redox probe. Coupled to a custom portable reader, the aptasensor showed a low LOD of 0.0032 U mL^−1^ for CA15-3 and was used for protein analysis in 30% serum samples and stepwise 5- and 4-fold diluted nipple discharge samples, demonstrating beneficial POC testing performance.

To improve assay’s sensitivity, the ECL immunosensor was designed to maximize signal gain at low CA15-3 levels by engineering both the luminophore carrier and the co-reactant accelerator as electrocatalytic nanohybrids [152]. Polyethyleneimine (PEI) was grafted onto bimetallic ZnNi-MOF nanospheres (ZnNi-MOF/PEI) to create a multifunctional L012-based ECL probe (ZnNi-MOF/PEI–L012@Ab_2_), where PEI served simultaneously as a scaffold for immobilizing the L012 luminophore and the detection antibody and as an intrinsic co-reactant, yielding a self-enhanced ECL label. In parallel, CeO_2_ nanorods functionalized with Pt nanoparticles (CeO_2_–Pt@Ab_1_) were employed as a highly efficient electrocatalytic co-reaction accelerator that decomposes H_2_O_2_ into abundant reactive oxygen species, further amplifying the ECL response in a “signal-on” sandwich configuration upon CA15-3 binding. This approach enabled impressive 5.75 × 10^−5^ U mL^−1^ CA15-3 detection underscoring how rational integration of MOF–polymer luminophore carriers and Pt-decorated oxide nanozymes can drive down LOD for mucin-type biomarkers in breast cancer diagnostics. Yet the overall complex and expensive reagents used make the assay less suitable for routine applications.

#### 5.6.3. CA 125

**Figure 12:**
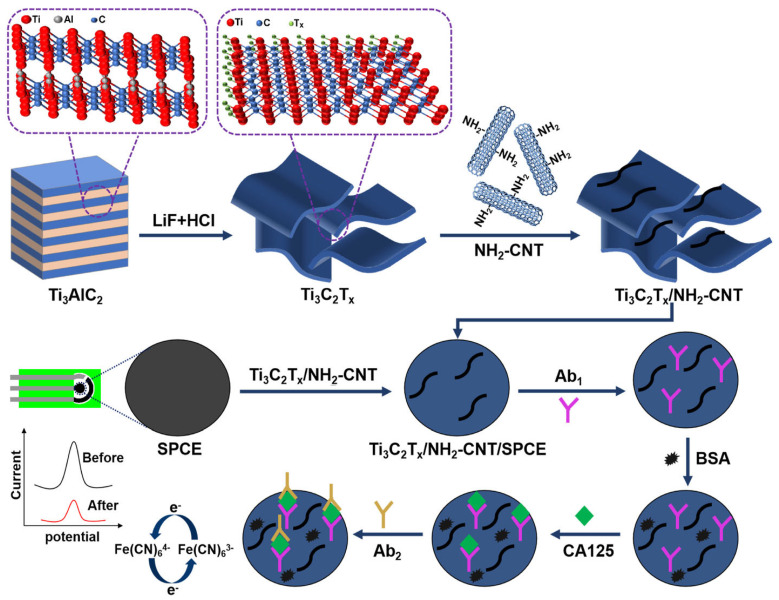
Schematic diagram of the construction and application of immunosensors for CA 125 based in MXenes and CNTs [154]. Copyright (2025) Elsevier, reprinted with permission.

A wide range of electrochemical and photoelectrochemical approaches for CA 125 detection has been explored to achieve the sensitivity required for early ovarian cancer diagnosis. A representative example is the photoelectrochemical (PEC) immunosensor based on a CdS/Bi_2_S_3_/NiS ternary sulfide heterostructured photocatalyst designed to amplify the PEC response towards CA 125 [153]. In this system, a CdS and Bi_2_S_3_ heterojunction promoted efficient separation of photogenerated charge carriers, while the narrow-bandgap NiS layer acted as an electron-conducting bridge, facilitating interfacial ET and markedly improving photoelectric conversion. Owing to synergistic architecture, the composite generated a photocurrent signal 14.6-fold higher than pure CdS. Anti-CA 125 antibodies were immobilized on this PEC enhanced scaffold via thioglycolic acid linkage, and the constructed immunosensor allowed down to 0.85 pg mL^−1^ CA 125 detection in serum samples. Complementary to this approach, a disposable electrochemical immunosensor for CA 125 was developed based on a Ti_3_C_2_T_X_-MXene/amino-functionalized carbon nanotube (NH_2_-CNT) composite, carbon SPEs, and ferricyanide as a redox indicator [154] (Figure 12). A conductive, high-surface-area Ti_3_C_2_T_X_:NH_2_-CNT hybrid amplified the electrochemical signal while the positively charged NH_2_-CNT mitigated MXene (Ti_3_C_2_T_X_) restacking and provided amino groups for covalent antibody immobilization. A chitosan film was used to improve adhesion of the nanocomposite to the electrode, yielding a mechanically stable, electrocatalytically active interface. The resulting immunosensor detected down to 1 mU mL^−1^ CA 125 and displayed selectivity, reproducibility, and long-term stability sufficient for serum samples analysis.

Covalent organic frameworks (COFs) in combination with a classic redox indicator were used to construct an electrochemical sandwich immunosensor for CA 125 with improved stability and high sensitivity [155]. A 2D epoxy-functionalized COF with ultra-high specific surface area was first assembled on a glassy carbon electrode, and capture antibodies were covalently attached to this epoxy-rich scaffold. A second COF (2D-COFBTT-DGMH) was used as a signaling tag, modified in situ with electropositive AuNPs to yield AuNPs@2D-COFBTT-DGMH/Ab_2_, modified by detection antibodies (Ab_2_) via Au–S bonding. In both cases, high surface density of antibodies was achieved. Importantly, the positively charged AuNPs@COF/Ab_2_ complex electrostatically attracted negatively charged ferricyanide, thereby boosting the faradaic signal from the redox indicator after CA 125 binding. The immunosensor enabled ultrasensitive 0.089 mU mL^−1^ CA 125 detection, yet it was never tested in any biological matrix. The same group constructed another sandwich immunosensor for CA 125 exploiting a COF-modified capture layer (another epoxy-functionalized COF) and an electroactive COF signaling tag bearing redox-active anthraquinone units decorated with AuNPs to form AuNPs@COFDAAQ-TFP/Ab_2_ [156]. The built-in electroactive reporter (the anthraquinone-based COF) eliminated the need for a soluble redox indicator, lowered LOD to 0.0067 U mL^−1^, and enabled sensor operation in 2% serum spiked with CA 125.

Selected examples of most advanced biosensors for mucins are summarized in Table 7.

### 5.7. Vascular Endothelial Growth Factor

Vascular endothelial growth factor (VEGF) belongs to the family of secreted glycoproteins that act as key regulators of angiogenesis, vascular permeability, and endothelial cell survival, primarily through binding to tyrosine kinase receptors such as VEGFR-1 and VEGFR-2 on endothelial cells [157,158,159]. Under physiological conditions, VEGF signaling is essential for embryonic vasculogenesis, wound healing, and tissue repair; however, in cancer it is frequently hijacked to sustain tumor growth, invasion, and metastasis by driving the formation of abnormal, leaky neovasculature, and by reshaping the tumor microenvironment [160,161]. Hypoxia-inducible factor (HIF)-mediated upregulation of VEGF is a canonical response to intratumoral hypoxia, and elevated VEGF expression has been documented in a wide spectrum of solid tumors, including colorectal, lung, breast, renal cell, hepatocellular, ovarian cancers, and glioblastoma [162], where it often correlates with increased micro-vessel density, aggressive clinical behavior, and poor prognosis [163,164].

**Figure 13:**
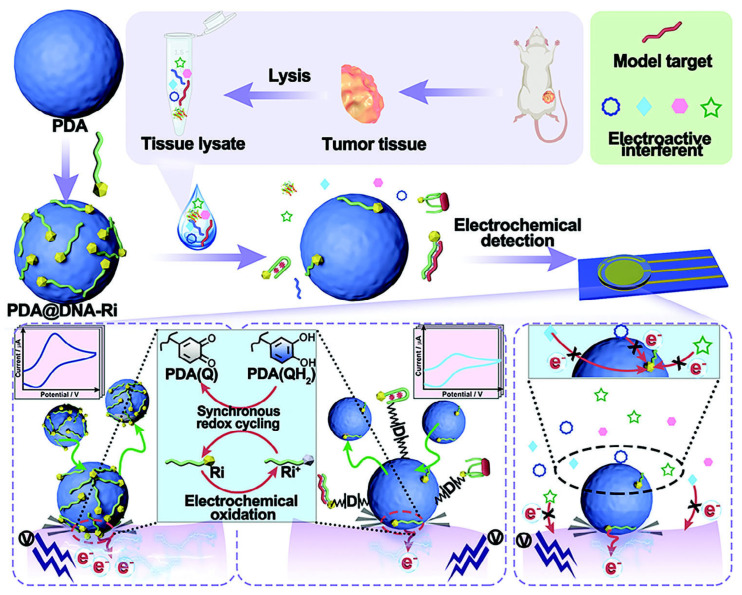
Schematic illustration of an electrochemical redox-cycling cascade nanosensor toward quantitative detection of VEGF in tumor tissue by loading biomarker-responsive redox indicator-labeled DNA probes (DNA-Ri, in green and yelllow) on the polydopamine nano-donor particles (PDA, in blue) for boosting synchronous ET, as proposed in [167]. Copyright (2024) Wiley, reprinted with permission.

These biological roles of VEGF, together with the fact that VEGF is readily detectable in circulation, have stimulated intense interest in developing analytical liquid biopsy platforms for VEGF quantification, including a growing number of electrochemical and electrocatalytic systems (Table 8). A label-free electrochemical immunosensor for VEGF was reported in which signal amplification relies on an electrocatalytically active AuNPs–reduced graphene oxide (AuNPs–rGO) nanocomposite [165]. The AuNPs–rGO layer provided a conductive, high-surface-area interface facilitating dense immobilization of anti-VEGF antibodies via carbodiimide coupling. Capacitive quantification of VEGF binding was performed by fast Fourier transform admittance voltammetry without the need for an external redox indicator, enabling LODs of 29.1 fg mL^−1^ on glassy carbon electrodes and 352 fg mL^−1^ on carbon SPEs. Another advanced electrochemical VEGF immunosensor built on a porous gold electrode fabricated by depositing a Ag/Au alloy onto an FTO substrate, followed by thermal annealing and electrochemical de-alloying to selectively remove silver and generate a high-surface-area, porous gold scaffold with excellent electrocatalytic properties (Figure 13) [166]. An immunosensor constructed by immobilizing VEGF-specific VHH (heavy-chain) antibody on this nanostructured Au surface enabled down to 0.05 pg mL^−1^ VEGF recognition probed by standard electrochemical techniques.

In a conceptually different approach, VEGF was targeted by an immobilization-free electrochemical nanosensor that amplified the biorecognition signal via a constrained ET cascade at the electrode surface [167]. In this system, electroactive nano-donors based on polydopamine (PDA) were labeled with a redox-indicator-tagged single-stranded DNA (Ri–ssDNA); upon specific VEGF binding, the ssDNA formed a duplex and dissociated from PDA, rendering the nanosensor response. At the redox tag oxidation potential, stochastic collisions of the PDA-based nanosensors with the electrode surface trigger synchronous redox cycling between PDA and the indicator, boosting the current output without requiring permanent capture or surface immobilization. Although demonstrated for multiple upregulated biomarkers, including miRNA-21 and ATP, the platform was particularly notable for VEGF, achieving a detection limit of 2.8 pM while strongly suppressing background contributions from physiological interferents through the restricted ET coupling.

Meanwhile, other groups have focused on surface-confined sensing architectures, in which the recognition element contributes to signal amplification. In this context, metal–organic frameworks (MOFs) have been employed as electrocatalytic supports for VEGF sensing. MWCNT SPEs were modified with an iron-based MOF MIL-100(Fe) and antibodies, yielding a MIL-100(Fe)/MWCNTs/SPE immunosensor with markedly enhanced electrochemical performance enabling 50 pg mL^−1^ VEGF detection alongside high selectivity in human serum comparable to ELISA [168]. Continuous electrochemical monitoring of VEGF was conducted with an electrochemical aptamer-based (E-AB) sensor exploiting the gold nanoparticle–DNA “pendulum” (GDP) mechanism [156]. VEGF-specific aptamer-GDP probes were assembled on a reduced-graphene-modified electrode. In the absence of VEGF, the GDP rapidly shuttled between solution and the electrode, producing fast current transients detected chronoamperometrically, while VEGF binding slowed the pendulum motion in a concentration-dependent fashion, lengthening the current decay time. This design yielded a drift-resistant, high-frequency readout suitable for real-time operation and enabled continuous, selective, and reversible VEGF detection (LOD = 0.0013 pM) in buffer and artificial urine.

In addition to isoform-agnostic VEGF sensing, several platforms have been designed specifically for VEGF_165_, the predominant 165–amino acid splice variant of VEGF-A and the main soluble, pro-angiogenic isoform driving pathological neovascularization in many solid tumors. VEGF_165_ retains high affinity for VEGFR-2, is abundantly secreted into the extracellular milieu and circulation, and is widely used as a functional readout of tumor-driven angiogenic signaling, making it a particularly relevant target for electrochemical biosensors [169]. A label-free electrochemical aptasensor for VEGF_165_ detection was designed using polydopamine–graphene (PDA–G) nanocomposite [170]. The PDA–G film was obtained via one-step self-polymerization of dopamine on graphene sheets and provided a conductive, high-surface-area interface enriched in catechol/amine (C–N) functionalities supporting aptamer immobilization. Impedimetric VEGF_165_ determination was achieved by monitoring Δ*R*_ct_ before and after VEGF_165_ binding under optimized conditions (pH 7.4, 37 °C, 30 min incubation). The aptasensor exhibited minimal matrix effects in diluted serum, enabling reliable quantification of endogenous VEGF_165_ levels in healthy donors (~28 pg mL^−1^).

**Table 8:** Analytical performance of selected electrochemical sensors for VEGF.

Strategy	Technique	Linear Range	LOD	Interference Studies	Ref.
GCE(SPCE)/AuNPs-rGO/MUA-MPA/Ab/BSA/VEFG	FFTAV	0.01–100 U mL^−1^	GCE: 29.1 fg mL^−1^ SPCE: 352 fg mL^−1^	PDGF, TGFβ, HAS, BMP, IGF-1	[165]
FTO/AuNPs/MAA/Ab/BSA/VEFG	EIS	0.1 pg mL^−1^–0.1 µg mL^−1^	0.05 pg mL^−1^	IFNγ, IGF-1, IL-2,	[166]
SPCE/PDA@DNA-Ri/VEFG	CV	10 pM–10 nM	2.8 pM	GTP, CTP, UTP	[167]
SPE/MWCNTs/MOF/Ab/HSA/VEFG	DPV	100–480 pg mL^−1^	50 pg mL^−1^	BSA, Glu, Cho	[168]
GCE/rGO/GDP/VEFG	CA	13 fM–130 nM	0.0013 pM	IgG	[156]
AuE/PDA-G/Apt/BSA/VEGF_165_	EIS	100–1000 pg mL^−1^	0.3 pg mL^−1^	BSA, IgG, Fibrinogen	[170]

Abbreviations: GCE: glassy carbon electrode; SPCE: screen-printed carbon electrode; rGO: reduced graphene oxide; MUA: 1,1-Mercapto Undecanoic acid; MPA: 3-Mercapto Propionic acid; FFTAV: FFT admittance voltammetry technique; PDGF: Platelet-Derived Growth Factor; TGFβ: Transforming Growth Factor; HAS: Hyaluronic Acid Synthase; BMP: Bone Morphogenetic Protein; IGF-1: Insulin-like Growth Factor 1; FTO: Fluorine-Doped Tin Oxide; AuNPs: gold nanoparticles; MAA: Mercaptoacetic acid; Ab: antibody; BSA: bovin serum albumin; EIS: electrochemical impedance spectroscopy; IFNγ: Interferon-gamma; IL-2: Interleukin-2; PDA: polydopamine; DNA-Ri: Ri-labeled DNA probes; CV: cyclic voltammetry; GTP: Guanosine triphosphate; CTP: Cytidine triphosphate; UTP: Uridine triphosphate; SPE: screen-printed electrodes; MWCNTs: multi-walled carbon nanotubes; MOF: metal–organic framework; HSA: human serum albumin; DPV: differential pulse voltammetry; Glu: Glucose; Cho: Cholesterol: GDP: gold nanoparticles DNA pendulum; CA: Chronoamperometry; IgG: immunoglobulin G; AuE: gold electrode; Apt: aptamer.

### 5.8. Carcinoembryonic Antigen

Carcinoembryonic antigen (CEA) is a heavily glycosylated cell-adhesion glycoprotein belonging to the immunoglobulin superfamily and expressed at very low levels in most healthy adult tissues yet becoming markedly upregulated in a range of epithelial malignancies [171,172]. In clinical practice, it is one of the oldest and most widely used serum tumor markers, particularly in colorectal cancer [173], where it is routinely measured for postoperative surveillance, detection of recurrence, and monitoring of therapy; typical reference values are <3 ng mL^−1^ in non-smokers and <5 ng mL^−1^ in smokers [174], though the exact cut-offs are assay- and lab-dependent. Elevated CEA levels are also frequently encountered in pancreatic, gastric, lung, breast and other gastrointestinal cancers, as well as in metastatic diseases, where they often correlate with tumor burden and more advanced stages [175,176].

Despite its limited specificity for early screening, this long-standing clinical utility and the existence of well-established decision thresholds have made CEA one of the central targets for analytical research. Within the period covered by this review, a large number of electrochemical sensing platforms have been reported for CEA detection, with majority of them focusing on nanostructured, biocatalytic, and electrocatalytic interfaces to achieve higher sensitivity, miniaturization, and POC applicability, rivaling the performance of routine immunoassays (Table 9).

**Figure 14:**
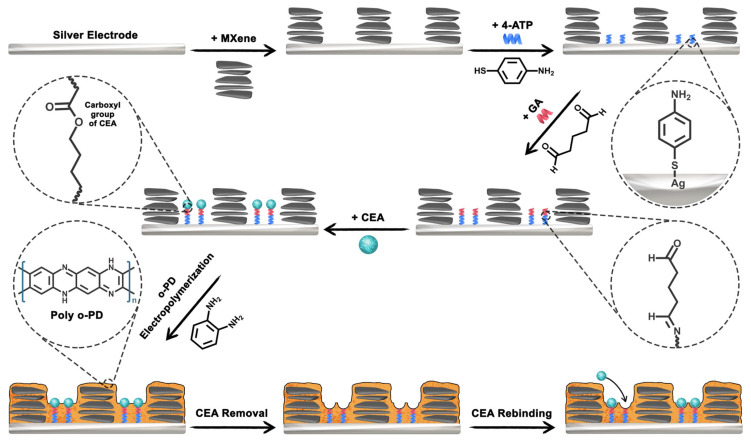
Stepwise schematic of the MIP biosensor fabrication process for CEA detection, illustrating modification of the silver working electrode via partial MXene surface coverage (in grey), and formation of 4-aminothiophenol (4-ATP) SAM, and CEA immobilization (cyan circles) on SAM using glutaraldehyde (GA), followed by surface imprinting via ortho-phenylenediamine (o-PD) electropolymerization, template CEA removal, and subsequent CEA rebinding [177]. Copyright (2025) ACS, reprinted with permission.

MXene-based Ti_3_C_2_T_X_ architectures were particularly actively used in electroanalytical platforms for CEA detection, benefiting both from high conductivity and rich surface chemistry of Ti_3_C_2_T_X_. A label-free impedimetric sensor in which Ti_3_C_2_T_X_ MXene was drop-cast onto disposable silver electrodes to create high-energy nanoareas for protein recognition, followed by CEA imprinting in o-phenylenediamine (o-PD) layer to form a CEA-specific MIP, which yielded a practical biosensor with a LOD of 9.41 ng mL^−1^ in 10% human serum and 10 min assay time, with features well aligned with POC monitoring of colorectal cancer patients (Figure 14) [177]. Carbon-nanotube-bridged Ti_3_C_2_T_X_ electrode arrays (MX@CNT) were fabricated by a template-assisted filtration method, generating a conductive network with very low background current and excellent electrocatalytic activity [178]. MX@CNT array was combined with a magnetic-bead alkaline phosphatase immunoassay (MB-aELISA) in situ, generating 1-naphthol as the electroactive product; the MX@CNT electrodes efficiently catalyzed oxidation of hydrolyzed 1-naphthol, with this amplifying the voltametric signal and enabling 1.6 pg mL^−1^ CEA detection in serum samples collected from colorectal cancer patients. In a related strategy, the intrinsic restacking of MXene was tackled by forming Ti_3_C_2_T_X_/MWCNT composites cast onto ITO and subsequently decorated with AuNPs to anchor anti-CEA antibodies [179]. MWCNTs acted as spacers preventing MXene aggregation and enhancing ET, while high loading of AuNPs improved antibody density and interfacial kinetics. The label-free immunosensor (BSA/Ab/AuNPs/MXene–MWCNTs–Nafion/ITO) showed a LOD of 0.015 ng mL^−1^.

CEA was also targeted with nanozyme-driven electrocatalytic platforms in which natural enzymes were replaced with more robust electrocatalytic nanomaterials. A Prussian-blue nanozyme immunosensor coupled a cascade enzyme–enzyme-like reaction using glucose oxidase (GOx) as a label in sandwich immunoassay and Fe–Co Prussian-blue analog (PBA) nanozymes with high peroxidase-like activity translating GOx activity producing H_2_O_2_ into electrocatalytic signal of H_2_O_2_ reduction [180]. The immunosensor detected 0.013 ng mL^−1^ CEA while simplifying assay operation and improving portability. In a related strategy, a label-free nanozyme electrochemical immunosensor for selective CEA detection integrated self-assembled peptide-templated Au/Cu bimetallic nanozymes and antibodies against CEA, with 5 mM H_2_O_2_ as added substrate [181]. Amphiphilic i3k peptides self-assembled into nanofibers, acting as biocompatible scaffolds for uniformly anchored Au/Cu nanoparticles (4:1 molar ratio), yielding enhanced peroxidase-like activity through synergistic Cu^2+^/Cu^+^–Au^0^ redox interactions, while Fc-functionalized chitosan film further mediated ET. This immunosensor offered a wide dynamic range (1.25–200 ng mL^−1^) and a LOD of 0.15 ng mL^−1^ in spiked serum, yet the necessity of external addition of H_2_O_2_ compromised assay’s suitability for POC testing. In addition to these peroxidase-mimicking systems, a conceptually elegant aptamer-gated nanozyme voltametric aptasensor was proposed, in which the catalytic activity of a Pt NPs/Fe-MOF nanozyme was switched by CEA binding [182]. In sensor design, Pt nanoparticles were loaded onto a Fe-based MOF (Pt NPs/Fe-MOF), yielding a nanozyme that efficiently catalyzed the oxidation of 1,2-diaminobenzene (OPD) to electroactive 2,3-diaminophenazine (DAP), reducible at the electrode surface. The CEA aptamer was adsorbed on the Pt NPs/Fe-MOF surface and sterically blocked the catalytic sites, which abolished the DAP signal. Addition and binding of CEA to the aptamer led to displacement of the aptamer from the nanozyme and recovery of its peroxidase-like activity, thus turning the electrochemical signal “on”. This aptamer-driven nanozyme platform afforded 0.125 pg mL^−1^ CEA detection but required adjusting samples’ pH to pH 4.0 and externally added 25 mM H_2_O_2_ and 2 mM OPD.

**Figure 15:**
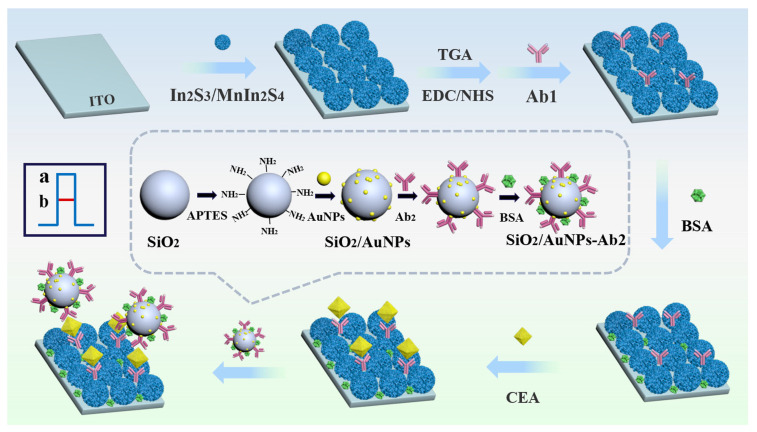
Schematic display of the photoelectrochemical immunosensor based on In_2_S_3_/MnIn_2_S_4_ proposed in [184]. In_2_S_3_/MnIn_2_S_4_ heterostructures (in blue) are immobilized on the indium tin oxide (ITO) electrode, treated with thioglycolic acid (TGA), and modified with antibody 1 (Ab1) via N-Ethyl-N’-(3-dimethylaminopropyl)-carbodiimide/N-hydroxysuccinimide (EDC/NHS) coupling. Then, after treatment with bovine serum albumin (BSA), the electrode is exposed to CEA (yellow diamonds) and further to SiO_2_ particles (in grey) modified with gold nanoparticles (AuNPs, yellow circles) and secondary antibody 2 (Ab2). Copyright (2025) Elsevier, reprinted with permission.

Within the AuNP-centered format, a label-free electrochemical CEA aptasensor was constructed, in which the conventional electrode was replaced by the AuNP-loaded one, maximizing both the number of electroactive and aptamer immobilization sites; additionally, a copolymer of polycaprolactone and hydroxyethyl acrylamide (PCL-b-PHEAA polymer) layer was used for impedimetric signal amplification [183]. An aptamer–CEA–aptamer “sandwich” configuration assembled on this high-surface-area AuNP-modified surface effectively suppressed cross-reactivity, and label-free analysis of changes in interfacial charge-transfer resistance allowed down to 60 fg mL^−1^ CEA detection by EIS in clinical samples. In the alternative application, an ultrasensitive PEC immunosensor based on an In_2_S_3_/MnIn_2_S_4_ heterojunction for CEA detection and AuNPs as quenchers was reported (Figure 15) [184]. MnIn_2_S_4_ nanosheets were grown in situ on In_2_S_3_ to form a lattice-matched heterostructure with optimized band alignment for visible-light harvesting and efficient separation of photogenerated carriers, markedly improving the PEC performance of the photoanode. For signal transduction, SiO_2_/AuNPs–Ab_2_ conjugates served as antibody-bearing tags that, upon CEA binding, were captured on the electrode and effectively quenched the photocurrent; the PEC signal, thus, decreased with increasing CEA concentration, starting from 0.143 pg mL^−1^ CEA. AuNPs were further used in a voltametric immunosensor constructed by layer-by-layer assembly of sodium alginate (SA), AuNPs and a γ-MnO_2_/chitosan (γ-MnO_2_–CS) nanocomposite and antibodies on glassy carbon electrodes, with ferricyanide as a redox indicator [185]. The SA/AuNPs/γ-MnO_2_–CS assembly increased electroactive surface area, while γ-MnO_2_–CS enhanced ferrocyanide response and provided a biocompatible matrix for antibody immobilization. CEA binding resulted in decreasing ferrocyanide oxidation currents due to formation of the Ab–CEA immunocomplex on the nanostructured surface, which enabled 9.57 fg mL^−1^ CEA. AuNPs have also been exploited in multiplex configurations, in an ultrasensitive sandwich electrochemical immunosensor for the simultaneous detection of CEA and the lung cancer-associated biomarker YES1 [186]. Biosynthesized AuNPs were coupled to thiolated protein G to orient and immobilize antibodies against both targets. After proteins binding, sandwich was formed with a secondary antibody labeled with horseradish peroxidase. The multiplex sensor showed a LOD of 0.0034 ng mL^−1^ for CEA and negligible cross-reactivity in human plasma.

Alternative nanomaterial strategies include graphene-based approaches such as a dual-mode electrochemical/FET aptasensor for CEA exploiting the high carrier mobility and large interfacial area of rGO [187]. An anti-CEA aptamer was immobilized on an rGO channel, and both the transistor transfer characteristics and cyclic voltammetry were used to monitor assembly and target binding. The rGO-based FET allowed label-free electrical detection of CEA at 0.33 zM levels, with a response time of 2 min, with extended dynamic range, offering a scalable, low-cost rGO-based assay with both extreme sensitivity and broad linear range. Graphene-derived interfaces were used in a smartphone-integrated, label-free electrochemical immunosensor for POC detection of serum CEA [188]. Graphene oxide, carbon nanotubes, and CuO nanoparticles (CuONPs/CNTs/GO/SPCE) were sequentially deposited layer-by-layer on carbon SPEs, generating a nanostructured film with high surface area, improved conductivity and ample sites for antibody loading. CEA binding to antibodies resulted in the decreasing response for ferricyanide used as a redox indicator, enabling impedimetric detection of 0.08 ng mL^−1^ CEA, also in serum, all within a portable, smartphone-readable format.

Other electrocatalytic strategies for CEA detection focused on further improving assays’ sensitivity, by engineering sophisticated PEC and ECL architectures. Self-powered dual-electrode PEC aptasensor was designed, in which the BiVO_4_/ZnIn_2_S_4_ Z-scheme heterojunction acted as a photoanode and the AuNP/CuBi_2_O_4_ composite served as a photocathode [189]. Strong internal field and synergistic light harvesting between the two electrodes produced a photocurrent under white light, while an aptamer-triggered G-quadruplex/hemin DNAzyme catalyzed the formation of insoluble products that markedly suppressed the photocathode current upon CEA binding. This electrocatalytically amplified “signal off” aptasensor enabled a LOD of 0.021 pg mL^−1^ and was tested in serum samples. Within the related PEC framework, a 2D Z-scheme ZnIn_2_S_4_/g-C_3_N_4_ heterojunction as a photosensitive layer and BiVO_4_ as a photo quencher were used in PEC immunosensor [190]. The Z-scheme semiconductor interface promoted efficient separation and transfer of photogenerated carriers, boosting the baseline photocurrent, while BiVO_4_ competitively consumed electron donors and blocked their contact with the substrate, leading to strong photocurrent attenuation upon immunocomplex formation. This dual-electrocatalyst configuration yielded a LOD of 0.03 pg mL^−1^. Further, a triple-enhanced ECL immunosensor was constructed in which the luminophore tris(2,2′-bipyridine)ruthenium(II) was encapsulated within an Fe-based MOF (NH_2_-MIL-88(Fe)) and coupled to a 3D Au@MoS_2_ nanoflower substrate [191]. The MOF provided a porous, protective host that stabilized the Ru(bpy)_3_^2+^ emitter and enhanced its local concentration, while the Au@MoS_2_ nanoarchitecture contributed to abundant catalytic sites and favorable charge-transfer pathways. Detection sensitivity (LOD of 38.9 fg mL^−1^) was boosted through three concurrent enhancement mechanisms: amino-catalyzed ECL, reversible Mo^4+^/Mo^6+^ redox activity in MoS_2_, and AuNP-mediated promotion of interfacial ET. The sensor showed high stability and specificity in serum.

## 6. Commercial Biosensors for Cancer Detection and Future Perspectives

Despite a large number of reported aptasensors and immunosensors for a variety of tumor-related protein biomarkers of cancer circulating in blood or urea, only few of them are present in the market, such as for serum PSA and AFP detection, and they are not electrochemical sensors, but rather, they operate within optical ELISA formats. Substantial efforts devoted to the development of electrochemical aptasensors employing enzyme and nanozyme labels, known as e-ELASAs, an approach rivaling traditional ELISA for detection of cancer biomarkers, have not yet resulted in a visible commercial success. E-ELASA offers robust, inexpensive, and highly sensitive tools for the selective analysis of protein biomarkers in human serum. However, none of the existing e-ELASA systems has yet reached the clinical or POC biosensors’ market. Despite significant progress, the translation of e-ELASAs as well as e-ELISAs from the laboratory to commercialization remains challenging. It may be tempting to speculate that the primary obstacle is the lack of simple, fully integrated platforms suitable for POC settings.

For clinical laboratories and POC environments, simplicity of biosensor operation and result interpretation is indeed essential, and analytical results must be both rapid and reliable. These devices are intended to be handled by minimally trained personnel; therefore, protocols must be straightforward, avoiding multiple steps, complex strategies, or specialized reagents, while ensuring high reproducibility. Looking forward, emerging trends may accelerate translation by enabling more integrated and scalable implementations. These approaches can integrate biosensor platform within microfluidic systems for protein detection [192], particularly, within the lateral and vertical flow test devices [193]. The resulting POC testing devices may be wirelessly integrated via Bluetooth with cloud-based computational platforms, enabling immediate data analysis and diagnostic decision-making [194]. In this context, coupling of electrochemical POC tests with AI-assisted signal processing and decision-support tools, such as calibration curve referencing, drift correction, signal quality control, and multimarker pattern recognition, could enhance robustness and simplify data analysis in real-world settings. In parallel, further exploration of wearable formats and related technologies, such as textile-based electrochemical sensors and smartphone-linked readers, for interstitial fluid (ISF)-based analysis of protein biomarkers may enable longitudinal monitoring and decentralized screening while generating high-quality data streams for personalized risk stratification. [195]. Nevertheless, given the current state of clinical validation of serum, urine, and saliva biomarkers, it is anticipated that research involving wearable ISF biosensors for protein cancer biomarkers may require additional time to mature.

Long-term storage stability of e-ELASA and e-ELISA kits is another critical requirement for POC screening. However, most sensors reported in the literature do not assess this parameter or report only short-term stability, typically not exceeding one month. In addition, POC devices should ideally be capable of analyzing untreated samples, such as a single drop of whole blood, a feature that is still rarely achieved, as sample pretreatment is usually required.

However, a more fundamental limitation in translating electrochemical biosensors from the laboratory to the market lies in the limited clinical validation of liquid biopsy cancer biomarkers, which in turn hampers the clinical validation and translation of the corresponding biosensors and liquid biopsy assays. Thus, a crucial step toward the commercialization of any biomedical sensor or assay is the demonstration of clinical validity of both the biomarker and the biosensor, the absence of which significantly limits its translational value. In most published studies, the primary focus is placed on analytical performance, while clinical validation is considered beyond the scope of the work. Frequently, biosensors are designed to target serum analytes whose biomarker relevance has been demonstrated only in the tumor microenvironment but not validated as circulating targets in blood (Table 1). In contrast, when industry evaluates a sensor for clinical adoption, the key criterion is its performance in a specific clinical context, namely, its validated ability to correctly classify patients with and without disease [196], and such validated liquid biopsy sensors are scarce. Machine-learning algorithms are now increasingly applied to multi-biomarker data analysis to improve diagnostic accuracy [15], particularly in heterogeneous early-stage cancers, where single-biomarker approaches fall short [197]. This approach requires the development of sophisticated deep-learning models, similar to those applied to genomic data analysis for cancer stratification [198]. However, molecular machine-learning models in proteomics remain scarce [199], largely due to the limited number of reliable liquid biopsy trials. Upon confirmation of protein biomarker signatures in liquid biopsy trials, the adaptation of established biosensor platforms for their detection could significantly accelerate commercialization.

## Figures and Tables

**Table 1:** Some selected potential liquid biopsy protein biomarkers of cancer, whose expression in tumors was correlated with cancer status.

Protein Name	Types of Cancer	Liquid Biopsy FDA-Approved
Human Epidermal Growth Factor Receptor-2 (HER-2/*neu*)	Breast, stomach, ovary, uterine, colon, bladder, lung, cervix, and esophagus cancer	No
Prostate-Specific Antigen (PSA)	Prostate cancer, metastatic prostate cancer	Yes
EGFR	Non-small cell lung cancer, colorectal cancer, cell carcinoma, glioblastoma and subsets of breast, bladder, and ovarian cancers,	No
α-Fetoprotein (AFP)	Liver and hepatocellular carcinomas	Yes
Osteopontin (OPN)	Breast, prostate, colon, liver, and lung cancer, mesothelioma	No
Mucins and derivatives	Bladder, breast, colon, lung, prostate, pancreatic, and ovarian carcinomas	No
Vascular Endothelial Growth Factor (VEGF)	Found in various tumors in response to hypoxia but not in normal tissue	No
Carcinoembryonic Antigen (CEA)	Colon, breast, gastric, pancreatic and NSC lung cancer	No

**Table 2:** Analytical performance of selected electrochemical sensors for HER-2/*neu*.

Strategy	Technique	Linear Range	LOD	Interference Studies	Ref.
AuE/AuNPs/MPA7Cys/Fe_3_O_4_ NPs-PEG-Ab	DPV	0.01–10 ng mL^−1^ and 10–100 ng mL^−1^	1.0 pg mL^−1^	Serum samples	[73]
AuE/t-PEG-SAM/MB-Apt/HER-2/[Fe(CN)_6_]^4−/3−^	CV	1 pM–10 nM	1.0 pM	BSA, serum	[74]
GCE/SA DBP-AuNPs/Ab/HER-2/Apt-AuNPs-Hyd/AgNO_3_	CV	0.1 pg mL^−1^–10 ng mL^−1^	1.4 fM	SK-BR-3, MCF-7, MCF 10A, HeLa	[75]
GrE/NC/MBs-Ab/HER-2/Apt-cellulase	CC	0.1 fM–0.1 nM	0.1 fM	uPA, Thr, HSA, serum	[55]
GrE/NC/MBs-Nb/HER-2/Nb-cellulase	CC	0.1 fM–1 pM	0.1 fM	uPA, Thr, HSA, serum	[77]
PET_f_/AirB GrE/NC/MBs-Apt_1_/HER-2/Apt_2_-cellulase	CC	0.1 fM–10 pM	0.1 fM	uPA, Thr, HSA, serum	[78]
Electrochemical lateral flow test/SPE/NC/MBs-Apt_1_/Apt_2_-cellulase	CC	0.1 fM–10 pM	0.1 fM	uPA, Thr, HSA, serum	[79]
GrE(SPE)/MBs-Apt_1_(Ab)/HER-2/Apt_2_-G4/Hemin	CV/CC	0.1 fM–10 pM	Ab-Apt: 1 fM Apt-Apt: 10 fM	uPA, HSA, Thr, serum	[80]

Abbreviations: DPV: differential pulse voltammetry; MPA: 3-mercaptopropionic acid; Cys: cysteamine; t-PEG-SAM: thiolated polyethylene glycol self-assembled monolayers; MB: methylene blue; Apt: aptamer; CV: cyclic voltammetry; BSA: bovine serum albumin; GCE: glassy carbon electrode; SA DBP-AuNPs: self-assembled 2,5-bis(2-thienyl)-1H-pyrrole-1-(p-benzoic acid) monolayers on gold nanoparticles; Ab: antibody; GrE: graphite electrode; NC: nitrocellulose; MBs: magnetic beads; CC: chronocoulometry; PSA: prostate-specific antigen; Nb: nanobodies; HSA: human serum albumin; PET_f_: transparent polyethylene terephthalate film; AirB GrE: airbrushed graphite electrode; uPA: Urokinase plasminogen activator; Thr: Thrombin.

**Table 3:** Analytical performance of selected electrochemical sensors for PSA.

Strategy	Technique	Linear Range	LOD	Interference Studies	Ref.
SPAuE/SAM/Strep/*b*Ab/PSA/Apt-HRP/TMB	DPV	0.26–62.5 ng mL^−1^	0.66 ng mL^−1^	rPSA, NGAL	[97]
GrE/NC/MBs-Ab/PSA/Apt-cellulase	CC	0.5–50 ng mL^−1^	0.50 ng mL^−1^	-	[98]
SPE/MG/GQD/PTH/Anti-PSA-Ab/BSA/PSA	DPV	0.0125–1.0 ng mL^−1^; 1–80 ng mL^−1^	0.005 ng mL^−1^	CEA, CA125, CA153, BSA, UA, AA, Glu	[100]
GCE/PANI@Ti_3_C_2_-AuQD/Ab/BSA/PSA	DPV	2 fg mL^−1^–2 pg mL^−1^	0.61 fg mL^−1^	IgG, Strep, CEA, AFP	[101]
SPAuE/MX-PP/Ab/BSA/PSA	EIS	0.01–600 ng mL^−1^	4.96 × 10^−5^ ng mL^−1^	BSA, Glu, UA, AUM	[102]
SPE/HMCNs-FCA-Hb/Ab_1_/PSA/Ab_2_-TMB(Hb)	DPV	0.001–30 ng mL^−1^	0.11 pg mL^−1^	Trp, Cys, Glutamate, Lys, UA, BSA, Glu	[103]
AuE/SWCNTs/Peptide/PSA	R × t	1 × 10^−13^–1 × 10^−4^ µg µL^−1^	1 × 10^−7^ ng mL^−1^	BSA, IgG	[104]

Abbreviations: SPAuE: screen-printed gold electrodes; SAM: self-assembled monolayers; Strep: streptavidin; *b*Ab: biotinylated antibody; Apt: aptamer; HRP: horseradish peroxidase; TMB: 3,3′,5,5′-tetramethylbenzidine; DPV: differential pulse voltammetry, rPSA: recombinant PSA; NGAL: lipocalin-2; GrE: graphite electrode; NC: nitrocellulose; MBs: magnetic beads; Ab: antibody; CC: chronocoulometry SPE: screen-printed electrodes; MG: marigold flowers; GQD: graphene quantum dots; PTH: poly(thionine); BSA: bovin serum albumin; UA: Uric acid; AA: Ascorbic acid; Glu: glucose; GCE: glassy carbon electrodes; PANI: polyaniline; AuQD: gold quantum dots; IgG: immunoglobulin G; MX-PP: MXene modified with poly(3,4-ethylenedioxythiophene)/poly(styrene sulfonate); EIS: electrochemical impedance spectroscopy; AUM: normal artificial urine; HMCNs-FCA-Hb: ferrocene carboxylic acid and encapsulated hemoglobin; Trp: Tryptophan; Cys: Cysteine; Lys: Lysine; AuE: gold electrodes; SWCNTs: single-walled carbon nanotubes.

**Table 4:** Analytical performance of selected electrochemical sensors for EGFR.

Strategy	Technique	Linear Range	LOD	Interference Studies	Ref.
AuE/SiO_2_NS/Ab/EGFR/*b*Atp/Strep-ALP	LSV	1–1000 ng mL^−1^	0.06 ng mL^−1^	IgG, IgA, IgM, Lysozyme, Thr	[112]
SPCE/AgNWs/Apt/EGFR/[Fe(CN)_6_]^3−^/^4−^	CV	0.01 ng mL^−1^–1.0 μg mL^−1^	0.01 ng mL^−1^	PDGF	[113]
SPCE/3D-HCNSs/PdPtCuRu NPs/MB-ssDNA+Fc-ssDNA/EGFR-related bpwDNA A and B	EIS	1.0 × 10^−6^–1.0 × 10^−1^ nM	Bpwalker A and B: 3.4 fM and 2 fM	Wt DNA	[114]

Abbreviations: AuE: gold electrode; NS: Nanospheres; Ab: antibody; bApt: Biotinylated aptamer; Strep-ALP: Alkaline phosphatase modified with streptavidin; LSV: Linear sweep voltammetry; IgG: immunoglobulin G; IgA: immunoglobulin A; IgM: immunoglobulin M, Thr: Thrombin; SPCE: screen-printed carbon electrodes; NWs: Nanowires; CV: cyclic voltammetry; PDGF: Platelet-Derived Growth Factor; 3D-HCNSs: three-dimensional hollow carbon nanospheres–tetrametallic nanoparticle; PdPtCuRu NPs: Palladium–Platinum–Cupper–Ruteniun nanoparticles; MB: methylene blue;; bpwDNA A or B: circular bipedal DNA walkers A or B; EIS: electrochemical impedance spectroscopy; Wt DNA: Wild-type DNA.

**Table 5:** Analytical performance of selected electrochemical sensors for AFP.

Strategy	Technique	Linear Range	LOD	Interference Studies	Ref.
GCE/APDMAO/Apt_1_-MB/AFP/ConA-Ag NPs	DPV	10 fg mL^−1^–10 ng mL^−1^	3.41 fg mL^−1^	Lys, HSA, IgG, PSA, GLO, CEA, BSA, Thr	[122]
SPE/AuNPs-Ti_3_AlC_2_/Apt-MB/AFP	DPV	1.0–300 ng mL^−1^	0.05 ng mL^−1^	BSA, PSA, IgG, HGB, OVA	[123]
SPE/AuNPs/rGOCu_2_O-Apt/BSA/AFP	DPV	0.001–100 ng mL^−1^	1.77 pg mL^−1^	HSA, IgG, LDL, BSA	[124]
GCE/rGO-Au-Fe_3_O_4_/Apt-TB/BSA/AFP	SWV	0.1–500 ng mL^−1^	0.03 ng mL^−1^	CEA, BSA, IgG, PSA	[125]
GCE/PANI/CdTe/CdSe/DNA/MCH/AFP	DPV	1–10 μg mL^−1^	1 pg mL^−1^	MUC1, IL-6, CEA, IgG	[126]
GCE/GCN/PPy/Apt/AFP	ECL	1 × 10^−11^–1 × 10^−5^ µg mL^−1^	10^−11^ µg mL^−1^	BSA, Thr	[127]

Abbreviations: GCE: glassy carbon electrode; APDMAO: 3-aminopropyldimethylamine oxide; DPV: differential pulse voltammetry; Lys: Lysine; HSA: human serum albumin; IgG: immunoglobulin G; GLO: globulin, BSA: bovin serum albumin; Thr: Thrombin; SPE: screen-printed electrode; AuNPs: gold nanoparticles; Apt-MB: aptamer labeled with methylene blue; HGB: hemoglobin; OVA: ovalbumin; rGOCu_2_O-Apt: aptamer modified with reduced graphene oxide dopped with cuprous oxide; LDL: low-density lipoprotein; TB: toluidine blue; SWV: square wave voltammetry; PANI: polyaniline; MCH: 6-mercapto-1-hexanol; IL-6: Interleukin 6; GCN: graphitic carbon nitride; PPy: polypyrrole; ECL: electrochemiluminescence.

**Table 6:** Analytical performance of selected electrochemical sensors for OPN.

Strategy	Technique	Linear Range	LOD	Interference Studies	Ref.
ANME/AuNPs/4-MPBA/OPN/pMB/pDA	DPV	0.01–1000 ng mL^−1^	3 pg mL^−1^	MSLN, VEGF, PPZ, CPZ, Mg^2+^, Na^+^, DA	[133]
Au/SAM/Strep/*b*Ab/OPN	EIS	0.5−512 pg mL^−1^	0.171 pg mL^−1^	IL-8; CK-MB, VEGF	[134]
MGCE/Au@α-Fe_2_O_3_/Fe_3_O_4_/Apt_1_/OPN/Apt_2_/Cas13a	DPV	1.0 pg mL^−1^–10 ng mL^−1^	0.33 pg mL^−1^	NCL, HN-RNP-A1, AFP, Tau	[135]
GCE/Au/FeGdHCFNPs/oxGNPs/anti-OPN/OPN	DPV	5 × 10^2 1^–2 × 10^6^ pg mL^−1^	0.437 pg mL^−1^	ALP, Glu, DA, UA, Urea, VEGF, His, AA, BSA, HSA, Creatinine, Chloramphenicol	[136]

Abbreviations: ANME: acupuncture needle microelectrode; AuNPs: gold nanoparticles; 4-MPBA: 4-mercaptophenylboronic acid; pMB: poly methylene blue; pDA: poly dopamine; DPV: differential pulse voltammetry; MSLN: Mesothelin; PPZ: Perphenazine; CPZ: Chlorpromazine; SAM: self-assembled monolayer; Strep: streptavidin; *b*Ab: biotinylated antibody; EIS: electrochemical impedance spectroscopy; IL-8: Interleukin-8; CK-MB: creatine kinase; MGCE: magnetic glass carbon electrode; Apt: aptamer; NCL: Nucleolin; HN-RNP-A1: heterogeneous nuclear ribonucleoprotein A1; GCE: glassy carbon electrode; FeGdHCF NPs: iron–gadolinium hexacyanoferrate nanoparticles; oxGNPs: oxidized graphene nanoplatelets; ALP: alkaline phosphatase; Glu: glucose; UA: Uric acid; His: Histidine; AA: Ascorbic acid; BSA: bovin serum albumin; HSA: human serum albumin.

**Table 7:** Analytical performance of selected electrochemical sensors for all mucins.

Strategy	Technique	Linear Range	LOD	Interference Studies	Ref.
GCE/3D-CdCo-ONSs@AuNPs/Apt/BSA/MUC1	DPV	10 pg mL^−1^–100 μg mL^−1^	0.96 µg mL^−1^	UA, AA, HER2, CA125, CA199, CEA	[146]
GCE/Au/Apt_1_/MCH/Cas12a/CrRNA/MB (on signal) GCE/Au/Apt_1_/MCH/MNP-HP2- Cas12a/CrRNA/Apt_2_-MgAl-LDH@Fc-AuFe-MIL-101 (off signal)	DPV	10 fg mL^−1^–100 ng mL^−1^	5.97 fg mL^−1^ for MB 0.43 fg mL^−1^ for Fc	HER2, CA153, cTnI, Mb	[147]
g-C_3_N_4_/Au-luminol/Apt_1_/BSA/MUC1/ZnCuS-Apt_2_	ECL	1 × 10^−4^ ng mL^−1^–1 × 10^3^ ng mL^−1^	5 × 10^−5^ ng mL^−1^	AFP, CEA, HSA, Insulin	[148]
SPE/Ru-Pd@MXene-PANI-(Co-PP)/Au/ATP/Anti-MUC1/BSA/MUC1	EIS	1 fg mL^−1^–200 ng mL^−1^	0.28 fg mL^−1^	Survivin, AFP, Glu, BSA	[149]
SPCE/MWCNT/Au/4-ATP/nanoMIPs/CA15-3-activ-ated silica gel	SWV	1–100 U mL^−1^	0.14 U mL^−1^	HSA, IgG, CEA, Glu, AA	[150]
SPE/Au/BSA-PDA/Glut/Apt_CA15-3_/CA15-3/NU66-d@NR	DPV	0.01–1000 U mL^−1^	0.0032 U mL^−1^	CEA, AFP, IgG, IgM	[151]
GCE/CeO_2_-Pt-Streptavidin/Biotin-Ab_1_/BSA/CA15-3/Ab_2_@PEI-L012/ZnNi-MOF	ECL	0.0005–50 U mL^−1^	5.75 × 10^−5^ U mL^−1^	CEA, AFP, PSA	[152]
ITO/Cds/Bi_2_S_3_/NiS/Ab1/BSA/CA 125	PEC	1 pg mL^−1^–50 ng mL^−1^	0.85 pg mL^−1^	CEA, HE4, BSA, Creatine, UA, Glu, Cys	[153]
SPCE/CNT-NH_2_/Ti_3_C_2_T_x_/Ab_1_/BSA/CA 125/Ab_2_	DPV	1 mUmL^−1^–500 U mL^−1^	1 mU mL^−1^	PSA, CysC, LAM, ProGRP	[154]
GCE/TD-COF/Ab_1_/BSA/CA 125/Ab_2_/AuNPs@COF_BTT-DGMH_	DPV	0.00027–100 U mL^−1^	0.089 mU mL^−1^	NaCl, Ser, Glu, Cys, Arg	[155]
GCE/EP-COF_Dha-Tab_/Ab_1_/BSA/CA 125/Ab_2_-AuNPs@COF_DAAQ-TFP_	DPV	0.01–100 U mL^−1^	0.0067 U mL^−1^	KCl, DA, Glu, Fru, NaNO_2_, Man, Trp, Thr, Tyr, Cys, Arg	[156]

Abbreviations: GCE: glassy carbon electrode; 3D-CdCo-ONSs: three-dimensional cadmium-cobalt spindle oxide; AuNPs: gold nanoparticles; Apt: aptamer; BSA: bovin serum albumin; DPV: differential pulse voltammetry; UA: Uric acid; AA: Ascorbic acid; HER2: human epidermal growth factor receptor 2; CEA: carcinoembryonic antigen; MCH: 6-mercapto-1-hexanol; MNP-HP2: DNA hairpin probes immobilized on magnetic nanoparticles; crRNA: CRISPR RNA; MIL-8: chromium-based metal–organic frameworks; cTnI: Cardiac troponin I; ECL: electrochemiluminescence; Mb: Myoglobin; HSA: human serum albumin; SPE: screen-printed electrodes; PANI: polyaniline; SWV: Square-wave voltammetry; ATP: Adenosine triphosphate; Glu: Glucose; SPCE: Screen-printed carbon electrode; MWCNTs: multi-walled carbon nanotubes; PDA: polydopamine; IgG: immunoglobulin G; Glut: glutaraldehyde; IgM: immunoglobulin M; NU66-d@NR: NH2-UiO-66 functionalized with neutral red; Ab: antibody; MOF: metal–organic framework; ITO: Indium Tin Oxide; Cys: Cysteine; PEC: photoelectrochemical; CNT-NH_2_: carbon nanotubes with NH_2_ groups; CysC: Cystatin C; LAM: lipoarabinomannan; TD/EP-COF: epoxy-functionalized covalent organic framework; Fru: Fructose; Man: Manose; Trp: Tryptophan; Thr: Thrombin; Tyr: tyrosine; Arg: Arginine.

**Table 9:** Analytical performance of selected electrochemical sensors for CEA.

Strategy	Technique	Linear Range	LOD	Interference Studies	Ref.
AgE/Ti_3_C_2_T_X_/4-ATP/GA/CEA/Poly o-PD	EIS	10–100 ng mL^−1^	9.41 ng mL^−1^	CRP, Fibrinogen	[177]
SPCE-MX@CNT/MB/Ab_1_/BSA/CEA/Ab_2_-ALP	DPV	0.005–1.0 ng mL^− 1^	1.6 pg mL^−1^	Hb, AFP, HSA, Cys	[178]
ITO/Ti_3_AlC_2_/MWCNTs/Au/s-Ab/BSA/CEA	DPV	0.050–200 ng mL^−1^	0.015 ng mL^−1^	UA, Glu, AA, DA, BSA, PSA, AFP	[179]
SPE/Ab_1_/CEA/Ab_2_-AuNPs-GOx	DPV	0.020–100 ng mL^−1^	0.013 ng mL^−1^	HER2, PSA, cTnI, AFP	[180]
GCE/CS-Fc/i_3_k@Au/Cu/Ab/BSA/CEA	SWV	1.25–200 ng mL^−1^	0.15 ng mL^−1^	MUC1, IgG, MMP-2, MMP-7	[181]
GCE/MOF-Fe/PtNPs/Apt/CEA	DPV	0.003–15.0 ng mL^−1^	1 pg mL^−1^	Cys, PSA, BSA, AFP	[182]
AuE/SAM/AuNPs/SH-pt_1_/BSA/CEA/Apt_2_- COOH/PCL-b-PHEAA	EIS	100 fg mL^−1^–200 ng mL^−1^	6 × 10^−2^ pg mL^−1^	HER2, CYFRA21-1, cTnI	[183]
ITO/In_2_S_3_/MnIn_2_S_4_/TGA/Ab^1^/BSA/CEA/Ab_2_-AuNPs/SiO_2_	PEC	0.5 pg mL^−1^–100 ng mL^−1^	0.143 pg mL^−1^	IgG, Thr, NSE	[184]
GCE/SA/AuNPs/γ.MnO2-CS/Ab/BSA/CEA	DPV	10 fg mL^−1^–0.1 µg mL^−1^	9.57 fg mL^−1^	Vit C, Glu, Gly	[185]
GCE/Au/Prot-G/GA/Ab_1_/BSA/CEA/Ab_2_-HRP-AuNPs	DPV	0.1–50 ng mL^−1^	0.0022 ng mL^−1^	VEGF, BSA, Cys, GAPDH	[186]
iDE/rGO/PBASE/Apt/BSA/CEA	CV	0.1 fg mL^−1^–10 ng mL^−1^	0.33 zM	CYFRA21	[187]
SPCE/GO/CNTs/CuONPs/Ab/BSA/CEA	CV	0.1 ng mL^−12^–5.0 ng mL^−1^	0.08 ng mL^−1^	IgG, BSA, AFP, CYFRA21-1, CA 125	[188]
ITO/AuNP/CuBi_2_O_4_/MCH/Apt_1_/CEA/Apt_2_-G4/Hemin	PC	0.1 pg mL^−1^–10 ng mL^−1^	0.021 pg mL^−1^	AFP, BSA, IgG, PSA	[189]
FTO/ZnIn_2_S_4_/g-C_3_N_4_/AuNPs/Ab_1_/BSA/CEA/Ab_2_-BiVO_4_-	PC	0.0001–100 ng mL^−1^	0.03 pg mL^−1^	PSA, AFP, NSE, HIG, HSA	[190]
GCE/MoS_2_@Au-Ab_1_/BSA/CEA/Ab_2_-NH_2_-MIL88(Fe)/Ru(bpy)_3_^2+^	ECL	0.1 pg mL^−1^−100 ng mL^− 1^	38.9 fg mL^−1^	NSE, CYFRA21-1	[191]

Abbreviations: AgE: Silver electrode; 4-ATP: 4-Aminothiophenol; GA: Glutaraldehyde; o-PD: Ortho-phenylenediamine; EIS: electrochemical impedance spectroscopy; CRP: C-reactive protein; SPCE: screen-printed carbon electrode; MX: MXene; CNT: carbon nanotubes; MB: methylene blue; Ab: antibody; BSA: bovin serum albumin; DPV: differential pulse voltammetry; Hb: hemoglobin; HSA: human serum albumin; Cys: Cysteine; ITO: Indium Tin Oxide; MWCNTs: multi-walled carbon nanotubes: BSA: bovine serum albumin; UA: Uric acid; Glu: Glucose; AA: Ascorbic acid; DA: dopamine; SPE: screen-printed electrodes; AuNPs: gold nanoparticles; GOx: glucose oxidase; cTnI: Cardiac troponin I; GCE: glassy carbon electrodes; CS-FC: chitosan–ferrocene carboxaldehyde; i3k@Au/Cu: Au/Cu bimetallic nanozyme; SWV: square wave voltammetry; IgG: immunoglobulin G; MMP: matrix metalloproteinase; MOF: metal–organic framework; PtNPs: Platinum nanoparticles; Apt: aptamer; AuE: gold electrodes; SAM: Self-assembled monolayer; PCL-b-PHEAA: Copolymer of polycaprolactone and hydroxyethyl acrylamide; TGA: thioglycolic acid; PEC: photoelectrochemistry; Thr; Thrombin; NSE: neuron-specific enolase; SA: sodium alginate; Vit C: Vitamin C; Gly: Glycine; HRP: horseradish peroxidase; GAPDH: glyceraldehyde-3-phosphate dehydrogenase; iDE: interdigitated electrodes; rGO: reduced graphene oxide; PBASE: pyrene butyric acid N-hydroxy succinimide ester; CV: cyclic voltammetry; GO: graphene oxide; CuONPs: Copper oxide nanoparticles; MCH: 6-mercapto-1-hexanol; PC: Photocurrent; FTO: Fluorine-Doped Tin Oxide; HIG: human immunoglobulin G; NH_2_-MIL88(Fe): iron-based metal–organic framework; Ru(bpy)_3_^2+^: Tris(2,2′-bipyridine)ruthenium(II).

## Data Availability

No new data were created or analyzed in this study.
